# The PARN Deadenylase Targets a Discrete Set of mRNAs for Decay and Regulates Cell Motility in Mouse Myoblasts

**DOI:** 10.1371/journal.pgen.1002901

**Published:** 2012-08-30

**Authors:** Jerome E. Lee, Ju Youn Lee, Jarrett Trembly, Jeffrey Wilusz, Bin Tian, Carol J. Wilusz

**Affiliations:** 1 Department of Microbiology, Immunology, and Pathology, Colorado State University, Fort Collins, Colorado, United States of America; 2 Program in Cell and Molecular Biology, Colorado State University, Fort Collins, Colorado, United States of America; 3 Department of Biochemistry and Molecular Biology, University of Medicine and Dentistry of New Jersey–New Jersey Medical School, Newark, New Jersey, United States of America; University of California San Diego, United States of America

## Abstract

PARN is one of several deadenylase enzymes present in mammalian cells, and as such the contribution it makes to the regulation of gene expression is unclear. To address this, we performed global mRNA expression and half-life analysis on mouse myoblasts depleted of PARN. PARN knockdown resulted in the stabilization of 40 mRNAs, including that encoding the mRNA decay factor ZFP36L2. Additional experiments demonstrated that PARN knockdown induced an increase in *Zfp36l2* poly(A) tail length as well as increased translation. The elements responsible for PARN-dependent regulation lie within the 3′ UTR of the mRNA. Surprisingly, changes in mRNA stability showed an inverse correlation with mRNA abundance; stabilized transcripts showed either no change or a decrease in mRNA abundance. Moreover, we found that stabilized mRNAs had reduced accumulation of pre–mRNA, consistent with lower transcription rates. This presents compelling evidence for the coupling of mRNA decay and transcription to buffer mRNA abundances. Although PARN knockdown altered decay of relatively few mRNAs, there was a much larger effect on global gene expression. Many of the mRNAs whose abundance was reduced by PARN knockdown encode factors required for cell migration and adhesion. The biological relevance of this observation was demonstrated by the fact that PARN KD cells migrate faster in wound-healing assays. Collectively, these data indicate that PARN modulates decay of a defined set of mRNAs in mammalian cells and implicate this deadenylase in coordinating control of genes required for cell movement.

## Author Summary

Almost all cellular mRNAs terminate in a 3′ poly(A) tail, the removal of which can induce both translational silencing and mRNA decay. Mammalian cells encode many poly(A)-specific exoribonucleases, but their individual roles are poorly understood. Here, we undertook an analysis of the role of PARN deadenylase in mouse myoblasts using global measurements of mRNA decay rates. Our results reveal that a discrete set of mRNAs exhibit altered mRNA decay as a result of PARN depletion and that stabilization is associated with increased poly(A) tail length and translation efficiency. We determined that stabilization of mRNAs does not generally result in their increased abundance, supporting the idea that mRNA decay is coupled to transcription. Importantly, knockdown of PARN has wide ranging effects on gene expression that specifically impact the extracellular matrix and cell migration.

## Introduction

The poly(A) tail added to mRNAs during processing in the nucleus stimulates mRNA export and translation through its association with poly(A)-binding proteins. In contrast, the removal of the poly(A) tail renders transcripts translationally silent and is also the first step in decay of the majority of transcripts in eukaryotic cells [1]. As such, the process of deadenylation has the ability to profoundly influence cellular gene expression on multiple levels. Numerous mammalian deadenylases have been identified and characterized to varying extents. They fall into two enzymatic groups; the DEDD-type (including PARN, CAF1/CNOT7 and PAN2) which bear an Asp-Glu-Asp-Asp motif in their active site, and the Exonuclease/Endonuclease/Phosphatase (EEP) type (including CCR4/CNOT6, Nocturnin (CCRN4L) and Angel proteins (ANGEL1, ANGEL2)) [2]. Of the many known poly(A) shortening enzymes, the CCR4/NOT complex and PARN are by far the best studied. CCR4/NOT represents the major cytoplasmic deadenylase in yeast where it initiates decay of the majority of mRNAs [3]. The yeast CCR4/NOT deadenylase is a large complex and contains two subunits with deadenylase activity(Ccr4p and Caf1p) as well as several other factors including the NOT proteins (Not1p-Not5p). In mammals, there are five CCR4-like proteins (CNOT6, CNOT6L, CCR4N4L/Nocturnin, ANGEL1 and ANGEL2) and three CAF1-like proteins (CNOT7, CNOT8 and CAF1Z/TOE1). Of these, CNOT6, CNOT6L, CNOT7 and CNOT8 associate with the mammalian NOT proteins to form various CCR4/NOT complexes [4]. In mammalian cells, CCR4/NOT complexes have been implicated in both miRNA-mediated and AU-rich element (ARE) mediated mRNA decay mechanisms. CCR4/NOT is recruited to mRNAs by the miRNA-associated GW182 protein [5], and by the ARE-binding protein tristetraprolin (TTP/ZFP36) [6]. Thus, for mRNAs bearing certain sequence determinants, the CCR4/NOT class of related deadenylases has an important role to play in initiating mRNA decay.

On a biochemical level, PARN is perhaps the best understood deadenylase, in part because it is the predominant activity in mammalian cell extracts [7]. PARN is unique in being able to interact with both the cap and poly(A) tail [7]–[9] and has been linked with mRNA decay mechanisms in both the nucleus [10] and the cytoplasm [7], [11]. In the cytoplasm, PARN plays an important role in controlling gene expression during the maternal-zygotic transition in *Xenopus* [12] and during the DNA damage response in mammalian cells [11]. In the nucleus, PARN has been linked with decay of transcripts undergoing 3′ end formation following DNA damage [10] and is important for trimming the 3′ ends of snoRNAs during their maturation [13]. In addition, PARN interacts directly with RNA-associated factors including CELF1/CUGBP1 [14], PUM2 [15] and CPEB [16]. Very few *bona fide* mRNA substrates of PARN in mammals have been identified to date. The *Fos* and *Myc* mRNAs exhibit increased abundance following PARN KD in HeLa cells [10] and *Tnfa* transcripts are deadenylated in a PARN-dependent manner *in vitro* [17] but to our knowledge there is no published evidence for a direct effect of PARN on mRNA stability in living mammalian cells.

Because of the large number and diversity of deadenylase activities in mammalian cells it has been challenging to discern their individual roles and their global impact on cell function. It remains unknown whether each mRNA must be targeted by a specific deadenylase to achieve appropriate control of gene expression. The impact of deadenylase activity on mRNA decay rates, mRNA abundance and translation efficiency is also not clear. Previous attempts to address these questions using RNA interference approaches have suggested partially overlapping roles for the CCR4-like (CNOT6, CNOT6L) and CAF1-like (CNOT7, CNOT8) deadenylases [18]. Surprisingly, less than 2% of all mRNAs showed changes in abundance following depletion of either CNOT6/CNOT6L or CNOT7/CNOT8 [18], implying that there is either redundancy in function between the many different deadenylase enzymes and/or that changes in mRNA abundance are not a good measure of deadenylase impact. Global measurements of mRNA decay rates following knockdown of deadenylases are necessary in order to distinguish these possibilities.

In this study we aimed to shed light on the role of PARN deadenylase in C2C12 myoblasts by directly assaying global mRNA decay rates and mRNA abundances following knockdown of PARN. We identified a relatively small set of 40 mRNAs whose decay was reduced following PARN KD and independently verified this observation for four of these transcripts. For *Zfp36l2* mRNA we also showed that PARN knockdown induces elongation of the poly(A) tail and increased protein abundance. Enhanced translation efficiency in PARN KD cells was also observed for a reporter bearing the *Zfp36l2* 3′UTR. We conclude that PARN is directly required for deadenylation of *Zfp36l2* and almost certainly other mRNAs within the stabilized set. Interestingly, slower mRNA decay did not result in the expected increases in abundance for the majority of stabilized mRNAs. We attribute this to reduced transcription rates, supporting the recently established idea of coupling between mRNA decay and transcription [19]–[22]. We also investigated the effects of PARN depletion on cellular function. We determined that loss of PARN activity decreased the abundance of transcripts encoding factors linked with cell adhesion and cell movement; processes that require extracellular matrix (ECM) interactions. This led to the discovery that depletion of PARN enhances wound healing in C2C12 myoblasts.

## Results

**Figure 1:**
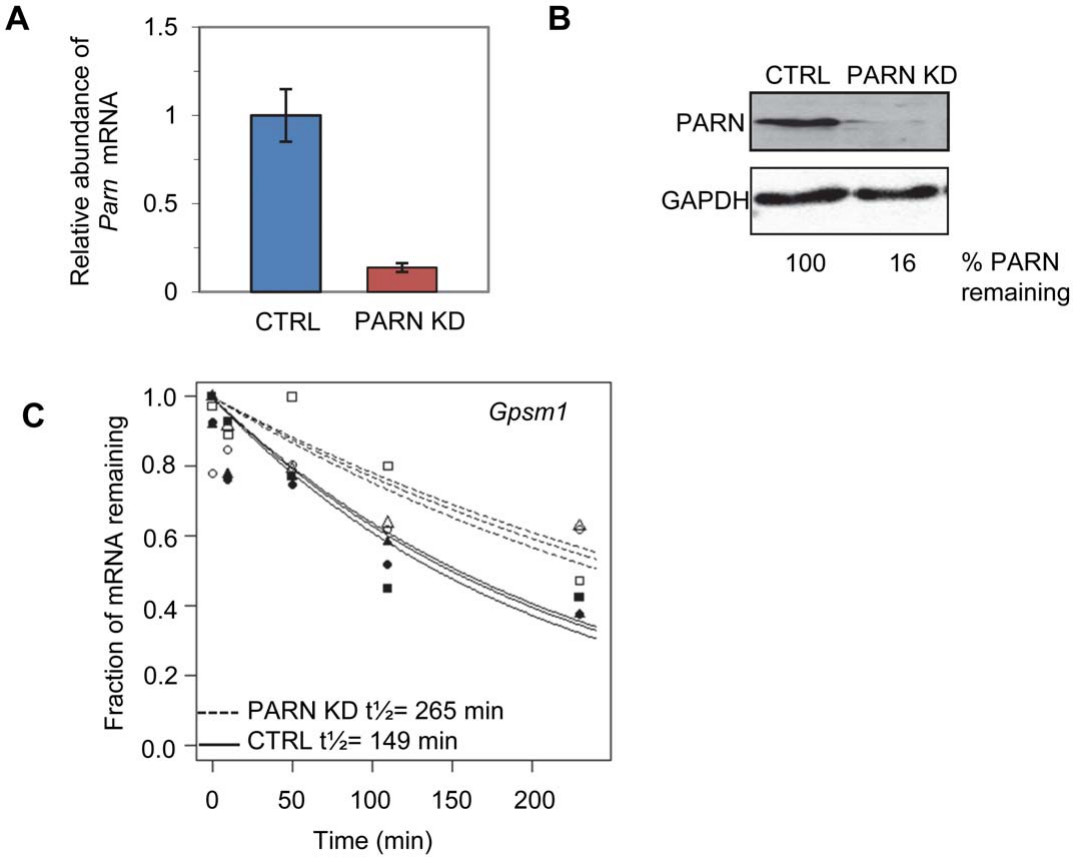
Determination of global mRNA decay rates in PARN knockdown cells. (A) Abundance of *Parn* mRNA was determined in CTRL and PARN KD cells by qRT-PCR and normalized to *Gapdh* mRNA. Error bars represent the standard deviation. (B) Abundance of PARN protein was determined in CTRL and PARN KD cells by western blotting and normalized to GAPDH protein. Relative expression of PARN is indicated below each lane. (C) Exponential decay curves generated from the microarray data for the *Gpsm1* mRNA in CTRL and PARN KD cells.

We used a lentiviral vector encoding an shRNA targeting the 3′UTR of murine *Parn* to generate a stable clonal C2C12 myoblast line with reduced expression of PARN (PARN KD). *Parn* mRNA and protein abundance were evaluated by qRT-PCR (Figure 1A) and western blotting (Figure 1B) in the PARN KD cell line and in a cell line generated with a control lentiviral vector lacking shRNA sequences (CTRL). The PARN KD cell line showed a robust reduction in PARN expression (Figure 1A and 1B).

We first wanted to assess mRNA decay rates in the PARN KD cells and compare them to those we obtained previously in the CTRL cell line [23]. Briefly, both cell lines were treated with Actinomycin D (Act-D) for 30 minutes and samples were collected at 0, 10, 50, 110 and 230 minutes after transcription inhibition. Total RNA was isolated from each sample and used to generate cDNA probes for hybridization to microarrays. The experiment was repeated in triplicate and three independent half-lives were generated for each transcript in each cell line by plotting the abundance at each time point and fitting to an exponential decay curve. As an example the half-lives for the *Gpsm1* mRNA in the two cell lines are shown in Figure 1C. Each half-life was considered reliable if the data fit well to the curve (p<0.05) and the 95% confidence interval was less than twice the half-life. We required that the half-life met these criteria for at least two of the three replicates. Both PARN KD and CTRL cells were assayed at the same time but analysis of the results from the CTRL cells was published previously [23].

**Figure 2:**
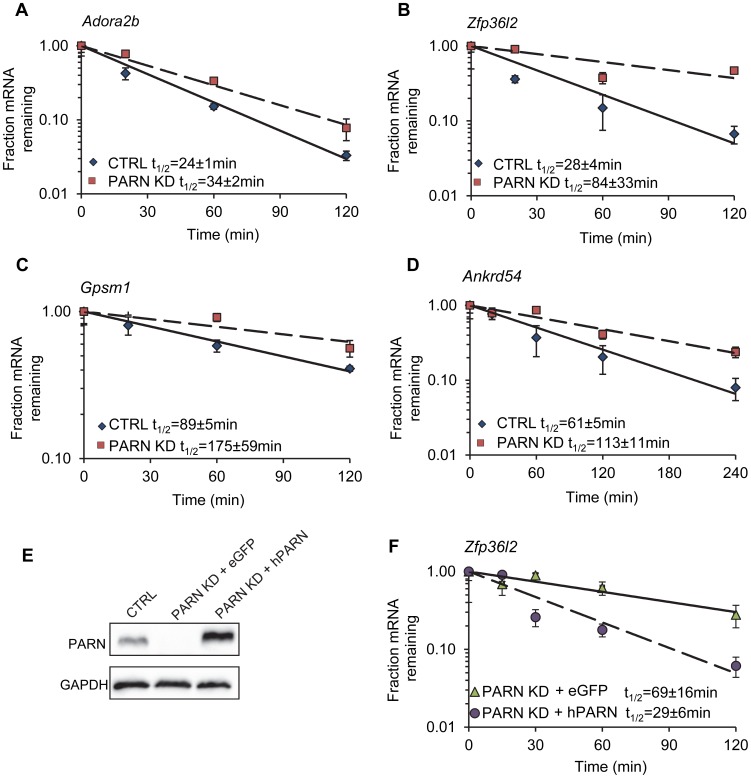
Independent assays validate the changes in decay in PARN KD cells and verify that stabilization is due to PARN depletion. mRNA decay rates for (A) *Adora2b*, (B) *Zfp36l2*, (C) *Gpsm1* and (D) *Ankrd54* were determined following Act-D treatment. mRNA abundance at each time point was assessed by qRT-PCR and normalized to that of *Gapdh* as a stable reference gene. Error bars represent the standard deviation derived from three independent experiments. Half-lives are reported with the standard error at 95% confidence intervals. (E) PARN KD cells were transfected with a control vector (eGFP) or with a construct encoding human PARN and PARN expression was verified by western blot using anti-PARN antibody with GAPDH as a loading control. (F) mRNA decay rates for *Zfp36l2* mRNA were evaluated by qRT-PCR in PARN KD cells expressing eGFP or human PARN. Errors represent the standard deviation. Half-lives are reported with the standard error at 95% confidence intervals.

Reliable half-lives were generated for 1581 mRNAs in the PARN KD cells (Dataset S1A; GSE35944). Although this dataset is somewhat smaller than that previously obtained for the CTRL cell line [7398 mRNAs; 23], it is nevertheless large enough to be informative. Overall, we obtained half-lives in both cell lines for 1389 mRNAs (Dataset S1B; GSE35944). Comparison of half-lives in CTRL and PARN KD cells allowed us to identify 64 transcripts that showed a statistically significant difference in decay rate between the two cell lines with 40 transcripts showing stabilization and the remaining 24 being destabilized (Table 1 and Table S1, respectively). To ascertain that the microarray analysis reflected true changes in mRNA decay rates, we assayed half-lives following Act-D treatment for four of the stabilized transcripts (*Adora2b*, *Zfp36l2*, *Gpsm1* and *Ankrd54*) by qRT-PCR (Figure 2A–2D). These transcripts were selected because they have relatively short half-lives (less than 2 hours) allowing us to assess their decay over a time frame that minimizes the toxic effects of Act-D on the cell. All four transcripts were significantly more stable following PARN knockdown, as predicted by the microarray analysis. Moreover, instability of the *Zfp36l2* mRNA was restored by transfection of an expression vector encoding shRNA-resistant human PARN demonstrating that stabilization was not caused by off-target effects of the shRNA on expression of unrelated genes (Figure 2E and 2F). Thus, we conclude that the PARN deadenylase influences decay rates of a subset of mRNAs in mammalian cells.

**Table 1:** Forty mRNAs are stabilized in PARN knockdown cells.

Gene ID	Gene symbol	Description	Control t_1/2_ (min)	PARN KD t_1/2_ (min)	p value	Fold Change t_1/2_	Fold Change Abundance
11541	*Adora2b*	Adenosine A2b receptor	80	189	2.70E-02	2.35	−1.48
231872	*Aimp2*	Aminoacyl tRNA synthetase complex-interactor	156	233	4.29E-02	1.5	+1.47
433693	*Akirin2*	Transcription regulator	75	106	2.05E-02	1.4	−1.10
56317	*Anapc7*	Anaphase promoting complex subunit 7	129	208	1.84E-02	1.61	−1.07
223690	*Ankrd54*	Ankyrin repeat-containing domain 54	112	201	7.00E-03	1.79	−1.26
68566	*Caly*	Calcyon neuron-specific vesicular protein	246	421	4.11E-02	1.71	+1.07
12569	*Cdk5r1*	Cyclin-dependent kinase 5, regulatory subunit p35	88	125	4.24E-02	1.42	−1.10
27886	*Dgcr14*	DiGeorge Syndrome Critical Region 14	97	133	2.88E-02	1.37	−1.04
353190	*Edc3*	Enhancer of mRNA Decapping	76	100	9.91E-03	1.32	−1.33
14082	*Fadd*	Fas (TNFRSF6)-associated via death domain	128	153	9.53E-03	1.2	−1.24
67998	*Fam134c*	Family with sequence similarity 134	88	151	2.66E-02	1.71	−1.54
14461	*Gata2*	Transcription factor	114	159	4.63E-02	1.39	−1.25
11692	*Gfer*	Growth factor, augmenter of liver regeneration	119	151	4.30E-02	1.27	1.20
13972	*Gnb1l*	G-protein beta subunit-like protein	73	145	1.94E-02	1.98	−1.20
67839	*Gpsm1*	G-protein signaling modulator 1	149	265	3.87E-03	1.78	−1.03
73338	*Itpripl1*	Inositol 1,4,5-triphosphate receptor interacting	179	251	4.57E-02	1.4	+1.18
16529	*Kcnk5*	Potassium channel, subfamily K	89	146	3.88E-02	1.63	−1.80
16534	*Kcnn4*	Calcium-activated potassium channel	103	158	2.19E-02	1.53	−1.17
118445	*Klf16*	Kruppel-like transcription factor	102	176	3.24E-02	1.72	+1.09
75660	*Lin37*	Lin37 homolog	107	240	4.32E-02	2.25	−1.52
232087	*Mat2a*	Methionine adenosyltransferase II, alpha	52	111	3.73E-03	2.12	−1.27
211577	*Mrgprf*	Mas-related G-protein coupled receptor	125	279	4.21E-02	2.24	−1.95
100609	*Nsun5*	Putative DNA methyltransferase	160	270	4.41E-02	1.69	+1.07
27275	*Nufip1*	Nuclear FMR1 interacting protein	68	99	3.25E-02	1.44	−1.06
54125	*Polm*	DNA-directed polymerase mu	220	340	4.56E-02	1.54	−1.16
56742	*Psrc1*	Proline/Serine rich protein regulated by p53	115	158	3.50E-02	1.37	−1.01
545622	*Ptpn3*	protein tyrosine phosphatase	145	262	2.07E-02	1.8	−1.62
170767	*Rfxap*	Regulatory factor X-associated protein	109	159	4.91E-02	1.46	−1.02
68867	*Rnf122*	RING-finger ubiquitin ligase	96	144	1.98E-02	1.5	−1.52
244668	*Sipa1l2*	Signal-induced proliferation-associated 1 like 2	134	193	4.82E-02	1.44	−1.69
56389	*Stx5a*	Syntaxin 5A	104	124	2.67E-02	1.19	−1.86
57752	*Tacc2*	Transforming acidic coiled-coil containing protein	109	159	4.62E-02	1.46	−1.05
103724	*Tbc1d10a*	GTPase activator TBC1 domain family	134	184	3.34E-02	1.37	−1.01
68276	*Toe1*	Nuclear deadenylase	71	102	6.02E-03	1.44	−1.19
22030	*Traf2*	TNF receptor-associated factor 2	71	98	3.60E-02	1.38	−1.12
106628	*Trip10*	Thyroid hormone receptor interactor 10	110	159	3.12E-02	1.44	+1.05
381560	*Xkr8*	X Kell blood group precursor related	117	194	1.92E-02	1.66	−1.01
232879	*Zbtb45*	Zinc Finger and BTB domain protein	96	129	1.41E-02	1.34	−1.15
69890	*Zfp219*	Zinc finger protein 219	134	193	1.37E-02	1.44	+1.04
12193	*Zfp36l2*	Zinc finger protein 36, C3H type-like 2	58	97	2.75E-02	1.67	−1.04

**Figure 3:**
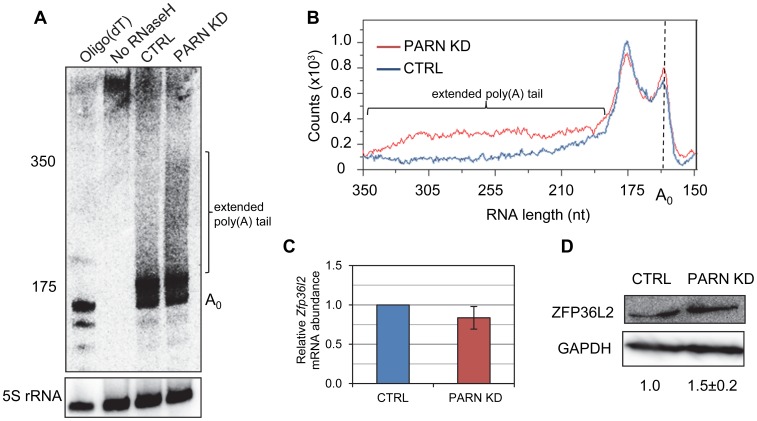
PARN modulates *Zfp36l2* poly(A) tail length to reduce expression of ZFP36L2 protein. (A) Total RNA from CTRL or PARN KD cells was treated with RNase H and an oligonucleotide that hybridizes within the 3′UTR of the *Zfp36l2* mRNA. The products were separated on a denaturing polyacrylamide gel and detected by northern blotting using a probe against the *Zfp36l2* 3′UTR. To generate an unadenylated marker, oligo(dT)_18_ was included in the RNase H treatment for the sample in the first lane. A probe against 5S rRNA was used as a loading control. (B) shows the profile of radioactive counts in the CTRL and PARN KD lanes with approximate size markers indicated on the x-axis. (C) *Zfp36l2* mRNA abundance was assessed by qRT-PCR in CTRL and PARN KD cell lines. Error bars represent the standard error of the mean derived from three independent experiments. (D) Western blot showing abundance of ZFP36L2 protein in CTRL and PARN KD cells. Expression was normalized to GAPDH. Relative abundances are indicated along with the standard deviation derived from three independent experiments.

Given that PARN is a deadenylase, we predicted that mRNAs stabilized by PARN KD would show effects on the length of their poly(A) tail. We investigated this possibility for the *Zfp36l2* mRNA using an RNase H/northern blotting approach. Briefly, total RNA isolated from CTRL and PARN KD cells was treated with an oligonucleotide and RNase H to induce cleavage ∼120 nt upstream of the poly(A) tail. After separation on a polyacrylamide gel followed by electroblotting, the 3′ fragment was detected using a radiolabeled probe complementary to the 3′UTR. As shown in Figure 3A and 3B, the poly(A) tail of *Zfp36l2* mRNA was clearly elongated in PARN KD cells compared to the CTRL cells. In fact, in the CTRL cells the vast majority of *Zfp36l2* mRNA had a surprisingly short poly(A) tail of just 20–30 nt. In the PARN KD cells the amount of *Zfp36l2* mRNA with a long poly(A) tail of up to ∼190 nt was two to three fold more than in the CTRL cells. This was not a general effect on all mRNAs as the β-Actin (*Actb*) mRNA showed no difference in poly(A) tail length between the two cell lines (Figure S1). Although abundance of *Zfp36l2* mRNA was similar in CTRL and PARN KD cells (Figure 3C), western blotting (Figure 3D) demonstrated a small increase in abundance of ZFP36L2 protein which would be consistent with enhanced translation resulting from the elongation of the poly(A) tail. We also saw evidence for increased abundance of ZFP36L2 protein by immunofluorescence (Figure S2).

**Figure 4:**
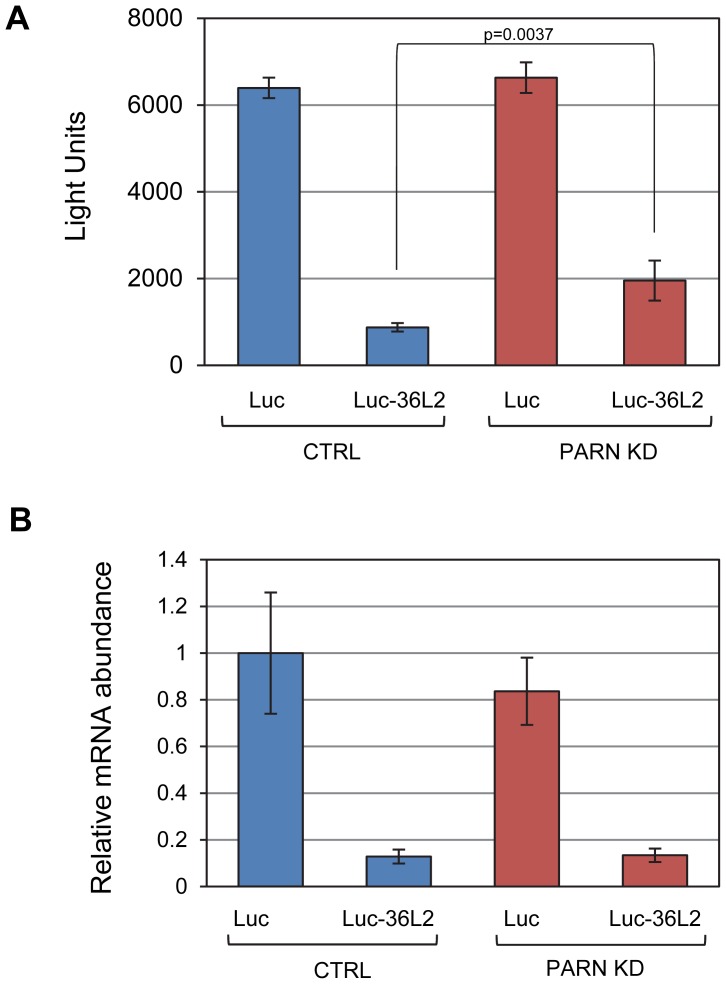
PARN acts through the 3′UTR of Zfp36l2 to repress translation. (A) Luciferase assays were performed in CTRL and PARN KD cells following transfection with empty pLightSwitch_3′UTR vector (Luc) or pLuc-36L2 which bears the Zfp36l2 3′UTR. Error bars are pooled standard deviations derived from three independent experiments. The asterisk indicates a statistically significant difference (p<0.002). (B) Abundance of Luc and Luc-36L2 mRNAs was determined by qRT-PCR and normalized to the abundance of *Gapdh* mRNA. Error bars represent pooled standard deviations derived from three independent experiments.

In order to determine whether the effects of PARN on the *Zfp36l2* mRNA are mediated by sequences in the 3′UTR we cloned the 3′UTR into a luciferase reporter construct (Luc-36L2) and measured luciferase activity following transfection into CTRL and PARN KD cells. The empty vector (Luc) was used as a control and gave very similar activity regardless of whether expressed in the CTRL or PARN KD cells (Figure 4A). Interestingly, the Luc-36L2 reporter produced significantly less luciferase activity than the Luc reporter in the control cell line suggesting that the sequences contained therein either repress translation or promote decay of the reporter mRNA. Importantly, PARN KD cells reproducibly exhibited a two-fold higher luciferase activity than the control cells (Figure 4A) and this was also seen when PARN was knocked down with a different shRNA (Figure S3) showing that this effect is PARN-specific. Interestingly, the clear increase in luciferase activity following PARN depletion is mediated predominantly by enhanced translation as there was little effect on abundance of either reporter mRNA in PARN KD cells (Figure 4B). The increase in luciferase expression is in the same range as the increase in abundance of endogenous ZFP36L2 protein in PARN KD cells (Figure 3D). Together these results indicate that the action of PARN on the Luc-36L2 reporter results in translation repression presumably through poly(A) shortening. Moreover, factors associated specifically with the 3′UTR of *Zfp36l2* mRNA are likely responsible for the effects of PARN on *Zfp36l2* gene expression. At this time we do not know what factor might be responsible for recruiting PARN, but the *Zfp36l2* 3′UTR does have AU-rich elements like those reported to bind proteins such as TTP/ZFP36; a protein that induces PARN-mediated deadenylation *in vitro* [17].

**Figure 5:**
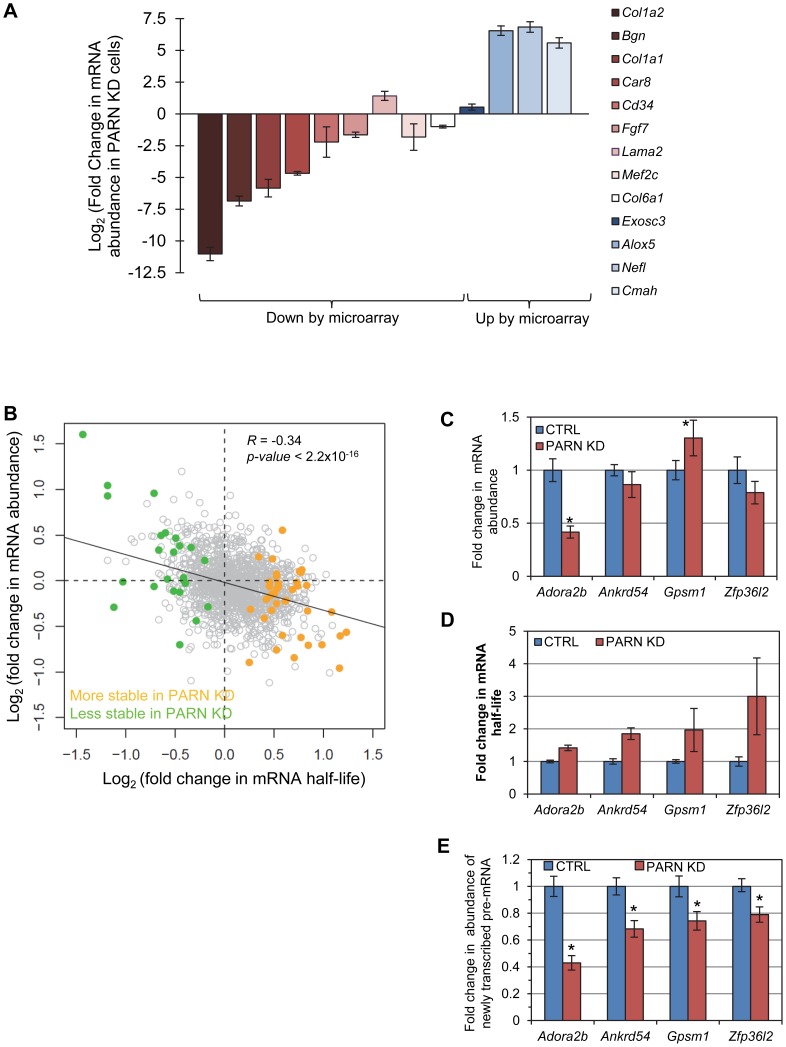
mRNA stabilization is not correlated with increases in mRNA abundance. (A) mRNAs that were predicted by the microarray to have significantly altered abundance were evaluated by qRT-PCR. Fold change in abundance in the PARN KD cell line as compared to the CTRL cells is shown. Error bars represent the pooled standard deviation from three independent experiments. Asterisks indicate statistically significant differences (p<0.05). (B) Scatter plot of fold change in abundance versus fold change in mRNA half-life for those mRNAs that showed significant changes in stability in PARN KD cells. The transcripts indicated in orange had increased stability following PARN KD, those in green were destabilized by PARN KD. Transcripts shown in grey had no significant change in half life. (C) The abundance of four transcripts that were stabilized following PARN depletion was evaluated in CTRL and PARN KD cell lines by qRT-PCR. The error bars represent the pooled standard deviation derived from four experiments. Statistically significant differences (p<0.05) are indicated by an asterisk. (D) The fold change in mRNA half-life for each mRNA is shown for comparison. This bar chart is derived from the same data as shown in Figure 2. (E) The abundance of newly transcribed pre-mRNAs was assessed by qRT-PCR and normalized to abundance of 7SL RNA. Statistically significant differences from the control (p<0.02) are indicated by asterisks.

The relatively small number of mRNAs affected by PARN at the level of mRNA stability precluded a meaningful analysis of Gene Ontology (GO) terms or sequences that might impacted by reduced PARN activity. Still, we did note that several of the stabilized transcripts encode proteins with roles in mRNA metabolism (*Toe1/Caf1z, Edc3, Zfp36l2, Dgcr14, Nufip1*) and transcription (*Gata2, Zfp219, Klf14*) indicating that PARN may influence gene expression at multiple levels and impact a wider range of genes. To investigate this possibility we used the 0 minute time point from the array experiments to estimate global mRNA abundances in CTRL and PARN KD cells. We found that of the 18,201 transcripts detected, 1199 showed a 1.5-fold or greater change in mRNA abundance in PARN KD cells (Dataset S2). Surprisingly, given that PARN KD was expected to increase expression of its target mRNAs, the majority (63.7%) of the affected mRNAs were down-regulated. We verified the abundance changes for several transcripts by qRT-PCR and found that of 14 mRNAs examined, all but one (*Lama2*) showed changes similar to those predicted by the array (Figure 5A). Moreover, there was generally a good correlation between the change predicted by the microarray and that observed by qRT-PCR in untreated cells although the qRT-PCR indicated changes of a greater magnitude than the array (Figure S4). This confirms that Act-D treatment did not globally affect our mRNA abundance measurements and that the 0 minute time point mRNA abundances are generally an acceptable indicator of relative differences in mRNA abundance between PARN KD and CTRL cell lines.

We next took advantage of the availability of both mRNA abundance and decay data to analyze the impact of changes in mRNA stability on overall mRNA levels. We were surprised to discover that for the 40 transcripts showing clear evidence for stabilization following PARN knockdown, there was generally only a small effect on mRNA abundance and in many cases abundance was reduced rather than increased (Figure 5B). There was a similar inverse correlation for the mRNAs that were destabilized (Figure 5B). In order to verify this observation, we measured the abundance of three transcripts that were stabilized by PARN depletion in proliferating myoblasts (Figure 5C). Interestingly, *Adora2b* mRNA (1.4-fold stabilized (Figure 5D and Figure 2A)) showed ∼2-fold reduced abundance in PARN KD cells, while *Ankrd54d* mRNA (1.85-fold stabilized) showed no statistically significant change in abundance (Figure 5C). In contrast, *Gpsm1* mRNA (1.96-fold stabilized) did show a small increase in abundance by this assay. As described earlier (Figure 3C), there was no significant change in abundance of the *Zfp36l2* transcript despite a ∼2.4-fold increase in stability. Taken together, these results strongly suggest the existence of coupling between transcription and decay for many transcripts such that changes in mRNA decay rate are compensated for by opposing effects on transcription [20]–[22].

In order to further support this idea we assessed the abundance of newly transcribed pre-mRNAs for each of the four stabilized transcripts. Briefly, C2C12 cells were labeled for a short time with 4-thiouridine (4sU) and total RNA was prepared. Newly transcribed 4sU-labeled RNAs were biotinylated and isolated on streptavidin beads. Pre-mRNAs were detected and quantified by qRT-PCR using one intronic primer and one exonic primer. As shown in Figure 5E, all four pre-mRNAs exhibited significantly reduced abundance in the PARN KD cells, consistent with slower transcription rates for these transcripts in this cell line. To summarize, each of the four mRNAs we evaluated showed increased stability following PARN KD (Figure 5D) but reduced levels of pre-mRNAs indicating reduced transcription (Figure 5E). This change in the relative balance of decay and transcription results in only small changes in mRNA abundance (Figure 5C).

GO analysis using DAVID [24] revealed that the transcripts whose expression was most affected by PARN shared some interesting features (Tables S2 and S3). In particular, amongst the down-regulated genes there was a significant enrichment of mRNAs encoding proteins required for blood vessel development, cell adhesion, cell motion and axon guidance (Table S2). This is supported by the observation that a large proportion (∼15%) of the down-regulated mRNAs encoded extracellular proteins including several collagens (*Col1a1, Col1a2, Col6a1, Col6a2, Col3a1, Col12a1*), biglycan (*Bgn*) and matrix metalloproteases (*Mmp19, Mmp2*). In contrast, the up-regulated mRNAs were more likely to encode components of large ribonucleoprotein complexes such as the ribosome and spliceosome (Table S3).

**Figure 6:**
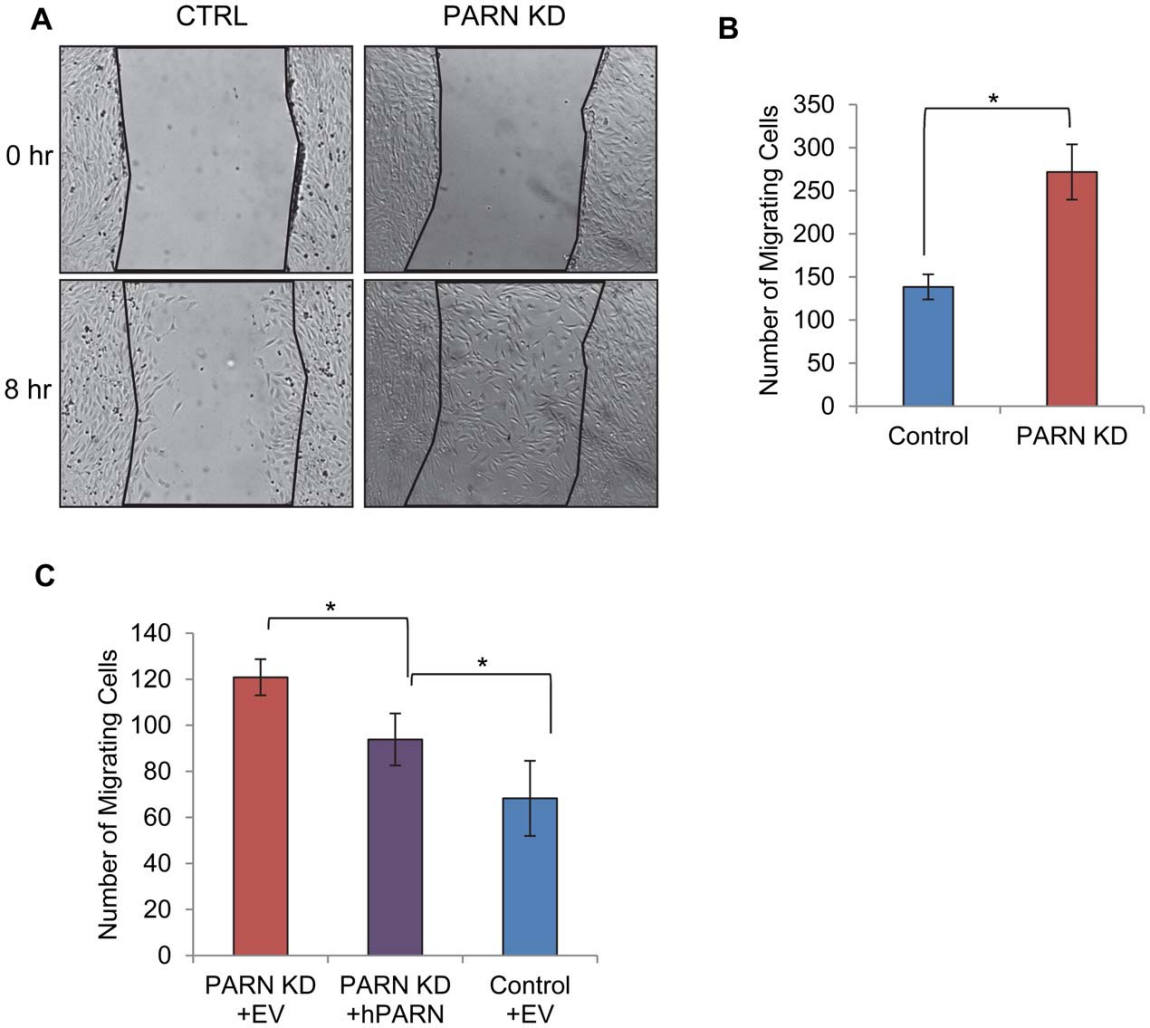
PARN knockdown results in enhanced wound healing. (A) CTRL and PARN KD were grown to confluency and displaced from the plate by scratching with a pipette tip. Wounds were allowed to heal over a period of eight hours and then the number of cells that had migrated into the wound was counted. (A) Brightfield images of the wounds in each cell line before and after incubation. (B) Graph showing the quantification of triplicate wound healing assays similar to those shown in (A). The error bars represent the standard deviation. (C) Graph showing the number of cells migrating into the wound in CTRL and PARN KD cells transfected with empty vector, and PARN KD cells transfected with a vector encoding human PARN. Error bars represent the standard deviation. All differences were statistically significant (*p<0.05).

Our GO analysis suggested that PARN knockdown might influence cell motility as cell movement requires extensive interactions with the extracellular matrix and is required for processes such as axon guidance and blood vessel development. We used a wound healing assay to investigate the ability of CTRL and PARN KD cells to migrate. Briefly, CTRL and PARN KD cells were grown to near confluence and then deprived of serum to prevent cell division. The monolayer was scratched to remove cells and incubated for eight hours to permit cells to migrate into the wound. Wound healing was assessed by counting the number of cells present within the boundaries of the wound. There was a clear increase in the wound healing capacity of PARN KD cells compared to the CTRL cells (Figure 6A and 6B) indicating that PARN KD cells migrate more rapidly. Moreover wound healing was restored to near normal levels following transfection of a plasmid encoding human PARN (Figure 6C). We conclude that PARN modulates processes required for cell motility in C2C12 myoblasts.

## Discussion

In this study we identified a set of mRNAs whose decay is dependent on PARN deadenylase. For one stabilized mRNA, *Zfp36l2*, we demonstrated that PARN-dependent regulation is mediated through sequences in the 3′UTR and that poly(A) tail length is increased following PARN KD. Depletion of PARN leads to increased ZFP36L2 protein abundance, but has negligible effects on mRNA abundance. Unexpectedly, we found that for the majority of affected transcripts mRNA stabilization slightly reduces mRNA abundance suggesting that mRNA decay rates are coupled to transcription. Finally, abundance of mRNAs encoding extracellular factors required for cell motility and adhesion was decreased by PARN knockdown and this observation led to the discovery that PARN KD cells migrate significantly faster than control cells in wound healing assays.

To our knowledge, ours is the first study to examine the role of the PARN deadenylase in mammalian cells, and the first to examine the global impact of depletion of an mRNA decay enzyme on mRNA decay rates. Our results suggest that while PARN directly impacts decay of relatively few transcripts, it has surprisingly wide-ranging effects on expression of over 1000 genes. This could reflect that some of the genes directly regulated by PARN have important roles in regulating transcription and other cellular processes, generating a knock-on effect. In addition, PARN-mediated deadenylation also clearly regulates translation efficiency (Figure 4), perhaps in some cases without altering mRNA decay rates. Any mRNAs whose poly(A) tail length is increased without a dramatic change in mRNA decay rate in PARN KD cells would not be detected by our analysis, although downstream effects of such regulation could be picked up as mRNA abundance changes. PARN is known to induce reversible deadenylation as a means to silence translation [16], however further experimentation will be required to distinguish targets regulated in this manner.

Despite the fact that poly(A) shortening is thought to enhance decay of mRNAs, we detected 24 mRNAs that were actually less stable following PARN KD. Some of these may be direct targets; perhaps when PARN is depleted a more aggressive decay pathway substitutes. However, given the wide-ranging effects of PARN KD on gene expression, we feel it is more likely that destabilization is an indirect effect of the PARN KD mediated by a factor(s) encoded by one of the stabilized transcripts (such as ZFP36L2, CAF1Z/TOE1 or EDC3). It also remains possible that some of these mRNAs are destabilized through off-target effects of the shRNA used to deplete PARN. Future experiments will aim to distinguish between these possibilities.

PARN KD stabilizes 40 of the 1389 mRNAs (2.9%) that we generated half-lives for in both cell lines. Remarkably, stabilization resulted in a decrease in abundance, or no significant change in abundance for the majority of these affected mRNAs (Table 1, Figure 3C, Figure 5B and 5C). This was seen in both the microarray and the qRT-PCR analyses. Although this seems counterintuitive, our results are actually very similar to recent observations on mRNA stability and abundance made in two closely related yeast strains [22]. These authors determined that as many as half of the evolutionary changes in mRNA degradation rates between *S. cerevisiae* and *S. paradoxus* were coupled to opposing changes in transcription rates. It was suggested that such coupling facilitates transient responses to environmental stimuli by enabling a more rapid return to basal expression levels. Additional studies, also in yeast, have established that mRNA decay rates are dependent on events that occur at the promoter [19]–[21] demonstrating that communication between transcription and mRNA turnover pathways exists. In yeast, some of this coupling has been attributed to the Rpb4/7 subunits of RNA polymerase II and to the CCR4-NOT complex, each of which have roles in both transcription and mRNA decay [19], [22]. Our data strongly imply that the cell attempts to compensate for loss of PARN by reducing transcription to maintain appropriate mRNA abundance. Interestingly, many stabilized transcripts appear to have slightly decreased abundance in PARN KD cells (Figure 5B). This overshoot suggests that the feedback mechanism is perhaps not highly accurate, or that it may additionally compensate for increased translation efficiency. Further investigation will be required to understand the mechanisms behind this feedback as so far there is no evidence that PARN modulates transcription directly, although it has been linked with mRNA 3′end processing events [10]. It is important to note that if extensive coupling of transcription and decay exists then mRNA abundance should be considered a poor indicator of both the magnitude and direction of effects on mRNA stability. This may explain why an earlier study found that depletion of CNOT6 and CNOT7 deadenylases had a relatively minor impact on overall gene expression [18].

Cell movement requires tightly controlled interactions between the cell and the ECM coupled with dynamic changes in the cytoskeleton. It can be described in three basic phases: Protrusion of the leading edge, adherence of the leading edge to the substrate and detachment of the cell body and trailing edge from the substrate [25]. Increased migration can be achieved by increased protrusion rate, by more efficient adherence to the substrate or by more rapid detachment from the substrate. While protrusion rate is primarily dependent on cytoskeletal dynamics, adherence and attachment can be influenced by cellular proteases or by the composition of the ECM. In general, those cell types that migrate most rapidly, such as leukocytes, have weaker interactions with the substrate whereas fibroblasts and myoblasts have stronger contacts and move more slowly [26]. Thus, the increased motility of PARN KD cells could be a direct result of alterations in the ECM caused by down-regulation of collagens, biglycan and other ECM components. Alternatively, increased motility could be due to altered expression of intracellular factors, such as TRIP10 and CDK5R1. The *Trip10/Cip4* mRNA (stabilized 1.44-fold by PARN KD) encodes Cdc42-interacting protein 4 which is localized to the leading edge of migrating cells and directly enhances cell motility through regulation of the actin cytoskeleton [27]. The *Cdk5r1* mRNA (stabilized 1.42-fold by PARN KD) encodes p35, an activator of the CDK5 kinase required for cell migration in neuroblastoma cells [28] and for myogenic differentiation [29]. Interestingly *Cdk5r1* mRNA is subject to extensive post-transcriptional control through both miRNA- and ARE-mediated mechanisms [28], [30]. Thus, increased expression of either CDK5R1 or TRIP10 proteins might directly enhance cell migration in PARN KD cells.

The fact that PARN KD enhances wound healing and cellular motility is interesting in light of previous observations that depletion of several RNA-binding proteins can affect wound healing in C2C12 cells; depletion of hnRNPD/AUF1, ELAVL1/HuR or IGF2BP2 impaired wound healing capacity and motility [31]. Another RBP, the zipcode binding protein IGF2BP1, has been implicated in regulating localized expression of mRNAs involved in cell adhesion in breast cancer cells [32]. Finally, the *Drosophila* 5′-3′ exoribonuclease Pacman (XRN1 in mammals) is required for normal wound healing [33]. These results suggest that factors important for cell motility may be subject to extensive post-transcriptional control.

Future studies will aim to further characterize the phenotype of PARN KD cells in order to decipher the mechanism by which cell motility is affected. It will also be interesting to determine whether PARN acts primarily on nuclear or cytoplasmic mRNAs. Finally, a high priority for future research is to uncover the mechanism by which changes in mRNA decay rates are signaled to the nucleus to influence transcription and to determine whether this is a global phenomenon in mammalian cells.

## Materials and Methods

### Cell lines and culture conditions

The mouse C2C12 myoblast cell line was obtained from the American Type Culture Collection (CRL1772). Two derivatives of the C2C12 cell line, CTRL and PARN KD, were cultured in Dulbecco's Modified Eagle's Medium (DMEM) containing 10% Fetal Bovine Serum (FBS), 1 µg/ml puromycin, 10 U/ml penicillin and 10 µg/ml streptomycin in 5% CO_2_ at 37°C. Cells were maintained at or below 70% confluency except during wound healing assays. Transfections were performed using Lipofectamine 2000 as described previously [34] and transfection efficiency was routinely in the 50–70% range. The CTRL cell line stably transfected with LKO1 vector was described previously [23]. The PARN KD clonal cell line was generated by puromycin selection following transduction with an shRNA-encoding lentivirus derived from the LKO1 vector [35]. This vector is described in more detail below.

### RNA isolation and microarray analysis of mRNA half-lives

The half-life experiment, microarray hybridization and analysis were all performed as described previously [23]. Briefly, CTRL and PARN KD cells were treated with Act-D (8 µg/ml) for 30 minutes prior to the start of the time course. Total RNA was isolated at several time points using TRIzol (Invitrogen) according to the manufacturer's directions. 300 ng of total RNA were used to generate labeled cDNA fragments for hybridization to Mouse Affymetrix Gene 1.0 ST Arrays following the manufacturer's protocol (GeneChip WT cDNA Synthesis Kit #900652 and #900720). Production of probes and hybridization was performed by the Colorado State University Genomics and Proteomics Core Facility. Half-life experiments were conducted in triplicate, with each time point hybridized to a single array.

For normalization of probe sets, we utilized the GC-bin method for background correction and applied median normalization by Affymetrix Power Tools (APT) with the ‘no adjustment’ option. Then, all probe set values were normalized to the 5th percentile value of all probe sets on the same array. Transcripts whose probe sets gave detection above background (DABG) p-value<0.05 in at least two out of three replicates at the 0 minute time point were considered expressed and used for subsequent analyses. A nonlinear least squares model [36] was used to calculate half-lives using the microarray data. A half-life measurement was considered reliable if it met both the following criteria: (i) the microarray data had a good fit to the nonlinear least squares model (p-value<0.05) and (ii) the 95% confidence interval for the half-life was less than two times the half-life. Transcripts with reliable half-lives in at least two of three replicates were selected for further analyses. The mRNAs whose half-lives were significantly different in PARN KD compared to CTRL C2C12 cells were selected based on the t-test (p-value<0.05). The datasets were deposited in the GEO database (GSE35944).

### Gene Ontology analysis

For functional analysis, lists of Gene IDs for those transcripts affected >1.5-fold were uploaded to the Database for Annotation, Visualization and Integrated Discovery (DAVID; [24]) along with the list of Gene IDs for all detected transcripts as Background. Functional clustering analysis was used to identify enriched groups of Gene Ontology (GO) terms. Clusters with enrichment scores of less than 1.3 (equivalent to a p-value greater than 0.05) were excluded.

### Quantitative Reverse Transcription PCR (qRT–PCR)

Total RNA was isolated using the TRIzol (Invitrogen) method as recommended by the manufacturer. All samples were treated with DNase 1 to remove genomic DNA. In experiments using samples from cells transfected with luciferase plasmids an additional step was employed to ensure effective removal of plasmid DNA. After the initial DNase treatment, RNA was treated with *EcoRI* and *EcoRV* to digest residual plasmid DNA and treated a second time with DNase 1. 1 µg of total RNA was reverse transcribed in the following conditions, according to the manufacturer's instructions: 35 mM Tris-Cl pH 8.3, 50 mM NaCl, 5 mM MgCl_2_, 5 mM DTT, 500 ng random hexamers, 10 U RNase Inhibitor, 1 µl Improm II Reverse Transcriptase (Promega). The resulting cDNA was used for qPCR with BioRad SYBR green supermix according to the manufacturer's instructions. A two-step amplification protocol was used in either a BioRad MyIQ, or a BioRad CFX96 instrument with annealing at 60°C for 30 seconds and extension at 95°C for 30 seconds for 40 cycles. mRNA abundances were normalized to the abundance of *Gapdh* mRNA except for experiments using 4-sU where 7SL RNA was used as a reference. Primer sequences are listed in Table S4.

### Plasmid construction

The pLightSwitch_3UTR vector was purchased from SwitchGear Genomics. The 3′UTR of *Zfp36l2* was PCR amplified from C2C12 myoblast cDNA using the following oligos (5′-GCTAGCCTCTCCATCTCCGACGACTG-3′ and 5′-CTCGAGTTGGGGGAAACTACAAAAC-3′). The resulting product was digested with *Xho*1 and *Nhe*1 and ligated into pLightSwitch_3UTR digested with the same enzymes to generate pLuc-36L2. The PARN expression clone bears the open reading frame of human PARN which was amplified using primers PARN1F (5′-CATGTCGACATGGAGATAATCAGGAGCAATTTT-3′ and PARN1R (5′-CATGGTACCTTACCATGTGTCAGGAACTTCAA-3′) and cloned between the *Xho1* and *Kpn1* sites of pcDNA3.1(-)(Invitrogen). It is not targeted by the murine PARN shRNA. The PARN targeting shRNA vector was generated by cloning annealed and kinased oligonucleotides (5′-CCGGGCGTGTGTGTTATTAACTAATCTCGAGATTAGTTAATAACACACACGCTTTTTG-3′ and 5′-AATTCAAAAAGCGTGTGTGTTATTAACTAATCTCGAGATTAGTTAATAACACACACGC-3′) into the *Age1* and *EcoR1* sites of the pLKO.1puro plasmid (a gift from R. Schneider; [35]). Oligonucleotide sequences were chosen from the Broad Institute's RNAi Consortium database (http://www.broadinstitute.org/rnai/trc). This particular shRNA targets the 3′UTR of murine PARN. The template used to generate the 5S rRNA probe was previously described [37]. In order to generate templates for probes against *Zfp36l2* and *Actb* mRNAs, total RNA was isolated from proliferating C2C12 cells, and the poly(A) tails were removed by RNase H treatment in the presence of oligo(dT)_18_. An RNA linker (Integrated DNA Technologies, Linker 3) was ligated to the 3′ ends of the RNAs using T4 RNA ligase treatment as described previously [38]. Ligated RNAs were subjected to reverse transcription using a specific primer complementary to the RNA linker (for details see [38]). The resulting cDNA which corresponded to the 3′ ends of the *Actb* and *Zfp36l2* mRNAs were then PCR amplified using the a primer complementary to the linker and an upstream oligo (ActB PAT 5′-CACTCCTAAGAGGAGGATGGTCGCGTC-3′ for actin and Zfp PAT 5′-CAGTTGGAGCACCGCGTGTG-3′ for Zfp36l2) and ligated into the pGemT-Easy vector (Promega). This process generated the pGemT-Zfp36l2 and pGemT-Actin plasmids which encode the 3′-terminal 300 nt of the *Actb* mRNA and the 3′-terminal 183 nt of the *Zfp36l2* mRNA.

### Western blotting

Whole cell lysate was prepared by lysis of cells in RIPA buffer (50 mM Tris-HCl (pH 7.4), 150 mM NaCl, 1.0% deoxycholate, 1% Triton X-100, 1 mM EDTA, and 0.1% SDS). 40 µg of each lysate was boiled in 6× protein loading buffer, resolved on a 10% SDS-polyacrylamide gel and blotted to PVDF membrane. PARN was detected using rabbit anti-sera (1∶20,000) [14]. ZFP36L2 was detected using rabbit polyclonal antibodies (Genway GWB-C5FC76). GAPDH (Chemicon mAB374) or Tubulin (Sigma Aldrich T5168) were used as loading controls (1∶20,000). Results were visualized using a BioRad Chemidoc system and quantified using QuantityOne software (BioRad). Reported values are a measure of the pixel density of the band of interest relative to the pixel density of the loading control (GAPDH or Tubulin). These ratios were normalized relative to control samples. Reported uncertainties are standard deviations.

### RNase H/northern blotting

10 µg of total RNA was incubated with 2 µM DNA oligo (ActB RNH 5′-AAGCAATGCTGTCACCTTCC-3′ for actin and Zfp RNH 5′-CGCGGTGCTCCAACTGTACCTA-3′ for Zfp36l2), heated to 95°C for three minutes and slow cooled to 4°C over a period of 30 minutes. RNaseH (7 units) and RNase Inhibitor (20 units) were added in the supplied reaction buffer (Fermentas Cat# EN0201). For generating poly(A) tail minus (A_0_) controls 100 ng/µl of oligo(dT)_18_ was included. Reactions were incubated at 37°C for 30 minutes. RNAs were then resolved on a 5% denaturing polyacrylamide gel (7 M urea, 1× TBE), and electroblotted to nylon membrane (Hybond-XL GE Healthcare) at 700 mA for 30 minutes in 1× TBE. Nucleic acids were immobilized by UV-crosslinking (Stratalinker). Membranes were pre-hybridized for 1 hour at 60°C in 25 ml hybridization buffer (50% formamide, 750 mM NaCl, 75 mM sodium citrate, 1% SDS, 0.1 mg/ml salmon sperm DNA, 1 mg/mL polyvinylpyrrolidone, 1 mg/mL ficoll, 1 mg/mL bovine serum albumin (BSA)). Membranes were then hybridized to radio-labeled RNA probe overnight at 60°C also in hybridization buffer. Blots were washed two times in 25 ml non-stringent wash buffer (0.1% SDS, 300 mM NaCl, 30 mM sodium citrate) and two times in 25 ml stringent wash buffer (0.1% SDS, 30 mM NaCl, 3 mM sodium citrate) for 20 minutes each time at 60°C. Membranes were exposed to storage phosphor screens and imaged on the Typhoon Trio Imager (GE Healthcare). Results were analyzed using ImageQuant software (GE Healthcare). α^32^P-labeled RNA probes were generated by *in vitro* transcription reactions as described below.

### *In vitro* transcription

Internally radio-labeled RNAs were generated by *in vitro* transcription reactions (20 U T7 or SP-6 RNA polymerase, 10 U RNase inhibitor, 40 mM Tris pH 7.9, 6 mM MgCl2, 10 mM DTT, 10 mM NaCl, 2 mM spermidine, 500 µM ATP, GTP, CTP, 50 µM UTP and [α-32P]-UTP(4.5 µCi/µl), 716 Ci/mmol) were carried out for 3 hours at 37°C using 1 µg of linearized plasmid DNA as template. For the RNase H/northern blot probes, the pGemT-Zfp36l2 construct was linearized with *SpeI* and transcribed with T7 RNA polymerase. The pGemT-Actin construct was linearized with *SacII* and transcribed with SP6 RNA polymerase. Transcription products were separated on a 5% polyacrylamide gel containing 7 M urea, excised and eluted overnight in 400 mM NaCl, 50 mM Tris-Cl pH 7.5, and 0.1% SDS at 22°C. RNA was precipitated and resuspended in H_2_O.

### Luciferase assays

C2C12 cells or PARN KD cells were transfected with a mixture of pEGFP-N1 (Clontech) and either Luc, or Luc36L2 plasmids. After 24 hours, the cells were trypsinized and collected in PBS. Coelenterazine (Promega) was added to a final concentration of 3 µM. Luciferase activities were measured in a Turner TD-20e Luminometer. Error bars represent pooled standard deviations derived from at least three independent experiments.

### 4sU-labeling and isolation of newly transcribed RNAs

Proliferating cultures of C2C12 myoblasts were treated with 4-thiouridine (200 µM; SIGMA) for 15 minutes. Following this labeling period, cells were collected in TRIzol and RNA was isolated according to the manufacturer's recommendation. Biotinylation and fractionation of RNAs was performed as described previously [39]. Briefly, this involved incubating 50 µg of total RNA with 100 µg of Biotin-HPDP in 100 mM Tris-Cl (pH 7.4) in the presence of 1 mM EDTA for 2 hours in the dark. An equal volume of chloroform and isoamyl alcohol (24∶1) was added to the biotinylation reaction and transferred to a Phase-Lock Gel tube (5 Prime), mixed by inversion, and centrifuged at full speed for 10 minutes at 4°C. RNA was precipitated in an equal volume of isopropanol in the presence of 0.5 M NaCl. The pellet was washed in 70% ethanol, resuspended in T.E. (10 mM Tris-Cl (pH 7.4) 1 mM EDTA), warmed to 65°C for 10 minutes and snap chilled on ice. RNA was mixed with an equal volume of streptavidin magnetic beads for 15 minutes at room temperature and loaded on an equilibrated MACS® Separation Column (Miltenyi Biotechnology). The beads were washed four times with 200 µl of wash buffer (100 mM Tris-Cl (pH 7.4) 10 mM EDTA, 1 M NaCl, and 0.1% Tween-20) at 65°C and twice more at room temperature. Labeled RNA was eluted with 100 mM DTT. Eluted RNA was precipitated as described above, washed and resuspended in 20 µl of ddH_2_O. 40 ng of eluted RNA was reversed transcribed using random hexamers. The resulting cDNA was used in qPCR to assess levels of pre-mRNA using primer pairs in which the reverse primer was complementary to an exon and the forward primer matched a region within the upstream intron. The pre-mRNA abundance was normalized to that of 7SL RNA.

### Wound-healing assays

C2C12 myoblasts (1.5×10^5^ cells) were seeded in 12-well dishes in growth media. Once cultures approached confluency, the monolayer was scratched with a 200 µl pipette tip. Cultures were washed with PBS, switched to low serum growth media (0.1% FBS), and imaged. After eight hours, cultures were imaged again. Margins of the scratch area were determined, and the number of cells migrating to the vacated area counted and graphed. Error bars represent the standard deviation from three experiments.

## Supporting Information

Dataset S1 Half-lives of mRNAs in PARN KD and CTRL cells. A: Half-lives of mRNAs in PARN KD cells. B: Comparison of half-lives in CTRL and PARN KD cells. GEO Accession: GSE35944.
(XLSX)

Dataset S2 mRNA abundance in PARN KD cells and CTRL cells at 0 minute time point.
(XLSX)

Figure S1 PARN KD does not affect the length of the *Actb* mRNA poly(A) tail.
(TIF)

Figure S2 Immunofluorescence shows elevated expression of ZFP36L2 protein in PARN KD cells.
(TIF)

Figure S3 PARN knockdown with an alternative shRNA also alleviates repression of translation mediated by the *Zfp36l2* 3′UTR.
(TIF)

Figure S4 Microarray and qRT-PCR analyses give similar results with regards to changes in mRNA abundance.
(TIF)

Table S1 Twenty-four mRNAs are destabilized in PARN KD cells.
(DOCX)

Table S2 Functional analysis of genes whose abundance was decreased >1.5-fold following PARN knockdown.
(DOCX)

Table S3 Functional analysis of genes whose abundance was increased >1.5-fold following PARN knockdown.
(DOCX)

Table S4 Gene Sequence Information.
(DOCX)
