# Supplementary figures and images for: The PARN Deadenylase Targets a Discrete Set of mRNAs for Decay and Regulates Cell Motility in Mouse Myoblasts
Source: PLoS Genet. 2012 Aug 30;8(8):e1002901. doi: 10.1371/journal.pgen.1002901 (PMC3431312; doi:10.1371/journal.pgen.1002901)

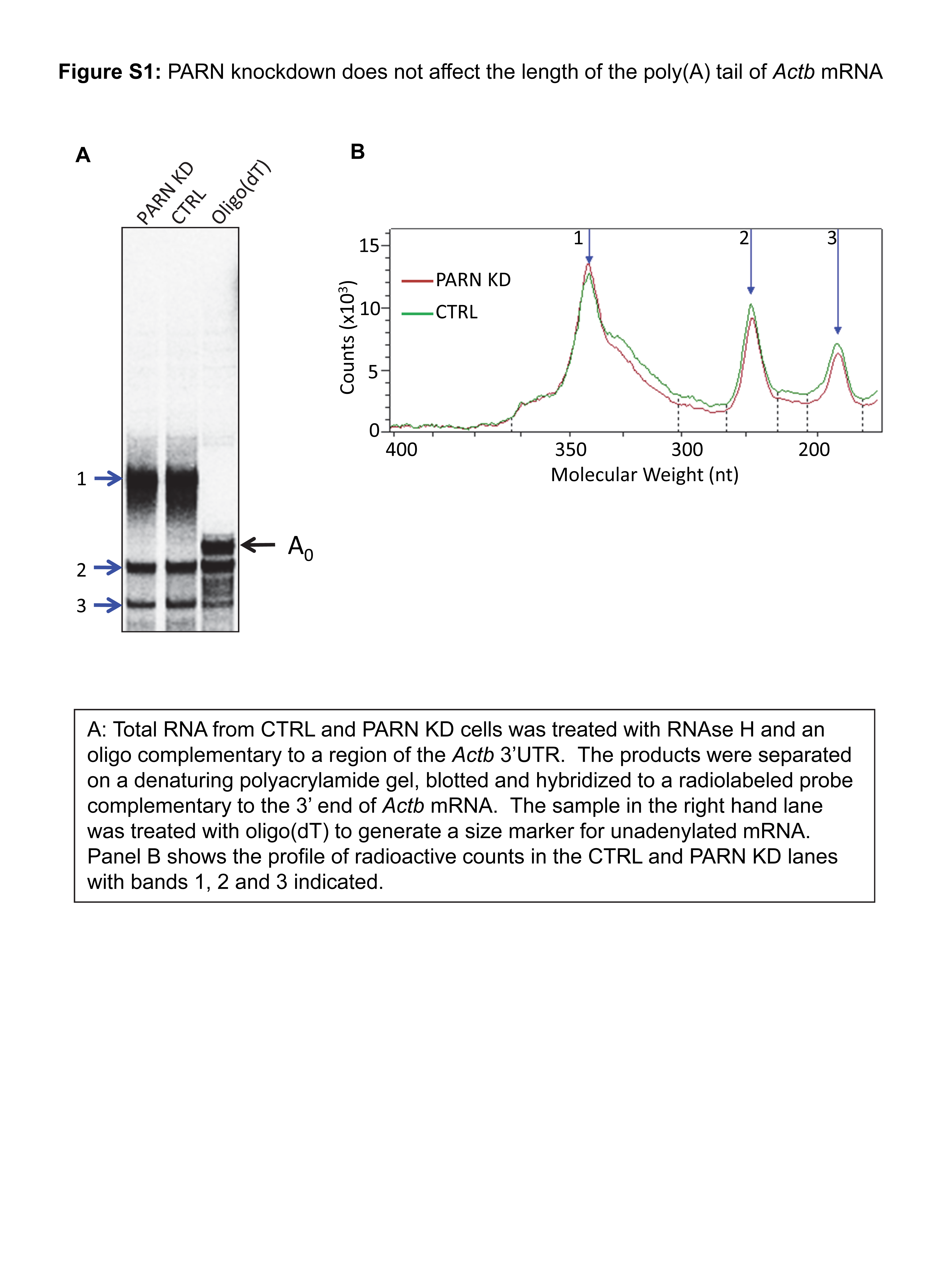

Supplement: Figure S1 — PARN KD does not affect the length of the Actb mRNA poly(A) tail. (TIF) [file pgen.1002901.s003.tif]

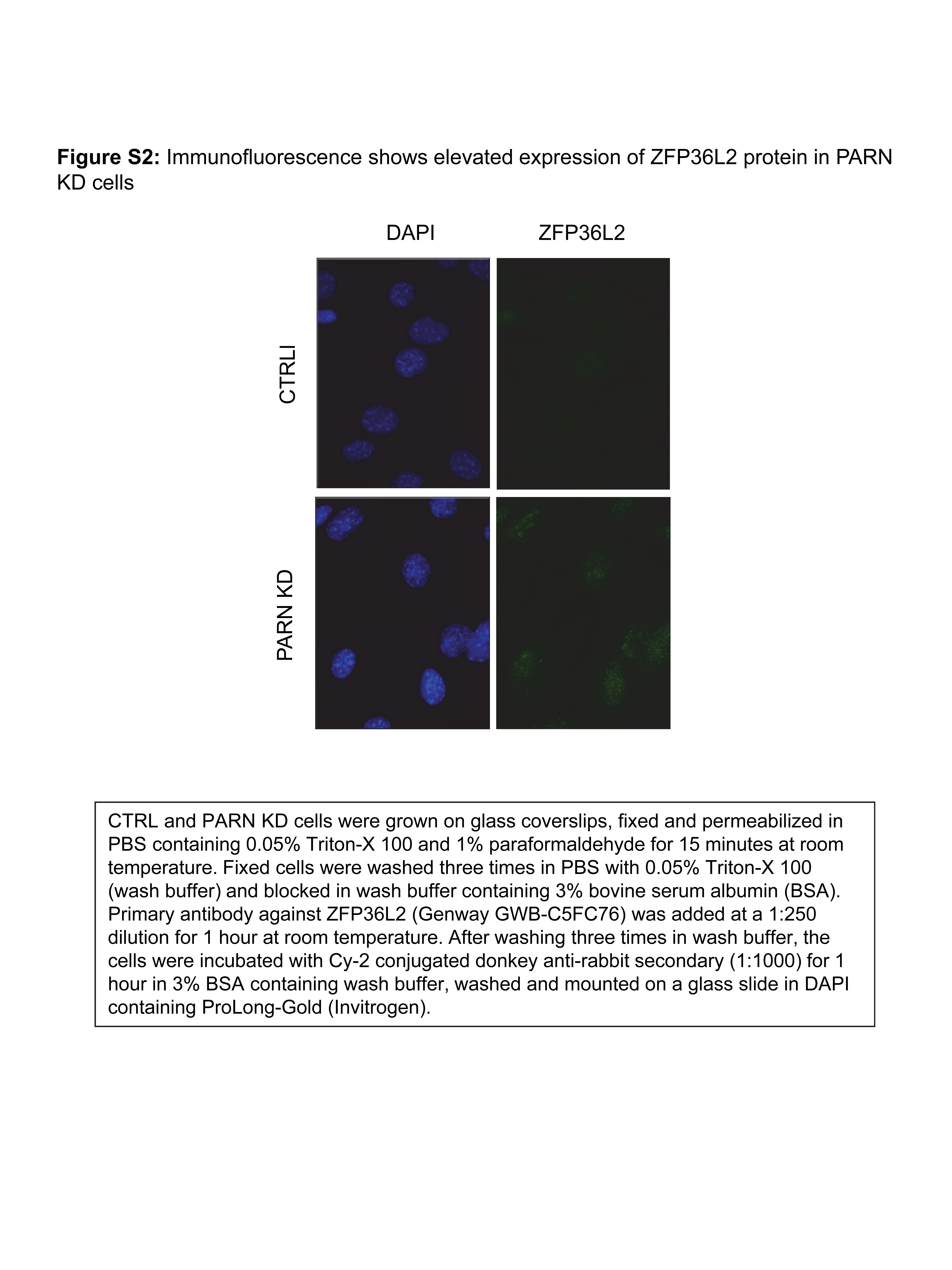

Supplement: Figure S2 — Immunofluorescence shows elevated expression of ZFP36L2 protein in PARN KD cells. (TIF) [file pgen.1002901.s004.tif]

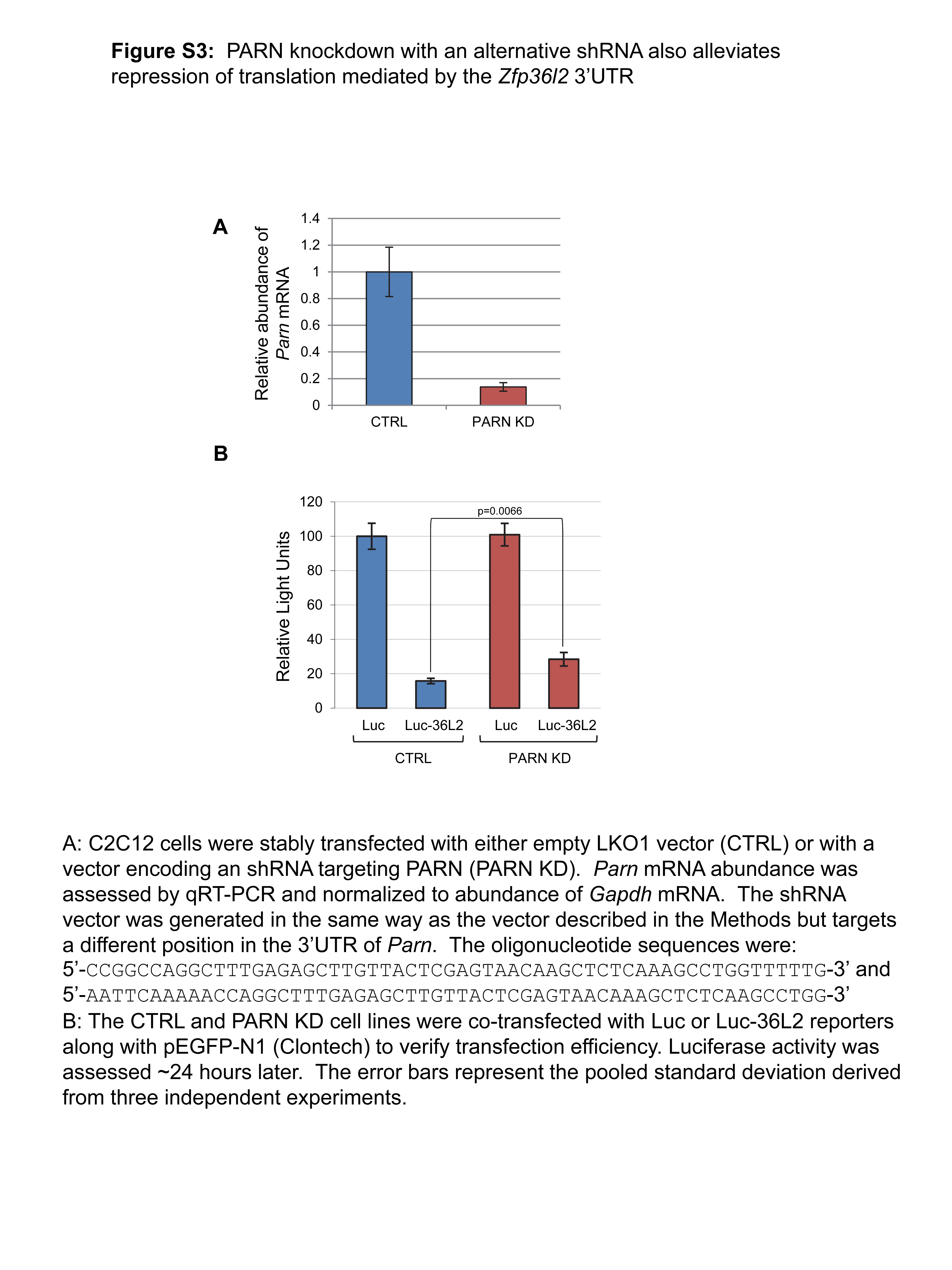

Supplement: Figure S3 — PARN knockdown with an alternative shRNA also alleviates repression of translation mediated by the Zfp36l2 3′UTR. (TIF) [file pgen.1002901.s005.tif]

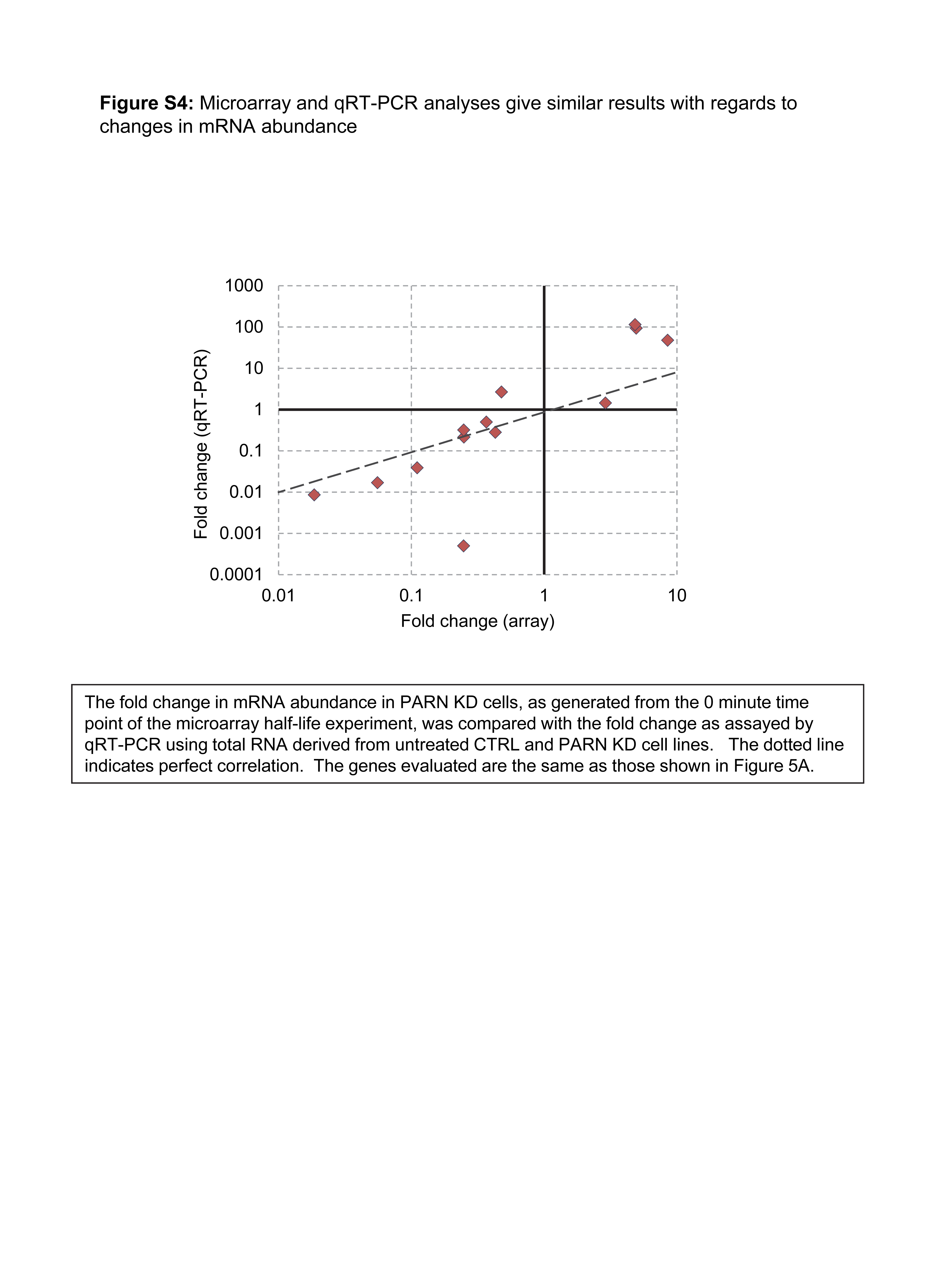

Supplement: Figure S4 — Microarray and qRT-PCR analyses give similar results with regards to changes in mRNA abundance. (TIF) [file pgen.1002901.s006.tif]
