# Supplementary material for: The PARN Deadenylase Targets a Discrete Set of mRNAs for Decay and Regulates Cell Motility in Mouse Myoblasts
Source: PLoS Genet. 2012 Aug 30;8(8):e1002901. doi: 10.1371/journal.pgen.1002901 (PMC3431312; doi:10.1371/journal.pgen.1002901)
Supplement: Table S1 — Twenty-four mRNAs are destabilized in PARN KD cells. (DOCX) [file pgen.1002901.s007.docx]

| **Gene id** | **Gene symbol** | **Description** | **Control t_1/2_ (min)** | **PARN KD t_1/2_ (min)** | **p value** | **Fold Change**  **t_1/2_** | **Fold Change Abundance** |
| --- | --- | --- | --- | --- | --- | --- | --- |
| 68277 | *2310057M21Rik* | Unknown function | 87 | 66 | 3.94E-02 | -1.32 | -1.02 |
| 109163 | *3010003L21Rik* | Unknown function | 214 | 138 | 2.02E-03 | -1.56 | 1.41 |
| 319719 | *4732471D19Rik* | Unknown function | 80 | 60 | 4.49E-02 | -1.33 | 1.03 |
| 70385 | *Ccdc99* | Coiled coil domain containing 99 | 136 | 83 | 2.20E-02 | -1.64 | -1.04 |
| 72170 | *Chchd4* | Mitochondrial intermembrane space import and assembly protein 40 | 97 | 70 | 6.61E-03 | -1.38 | 1.30 |
| 12747 | *Clk1* | CDC-like kinase 1 | 49 | 22 | 2.83E-02 | -2.16 | -1.22 |
| 70686 | *Dusp16* | Dual specificity phosphatase 16 | 92 | 66 | 4.97E-02 | -1.41 | 1.39 |
| 68969 | *Eif1b* | Eukaryotic translation initiation factor 1B | 134 | 101 | 1.41E-02 | -1.33 | 1.01 |
| 14234 | *Foxc2* | Forkhead transcription factor | 50 | 35 | 1.22E-03 | -1.44 | -1.08 |
| 23886 | *Gdf15* | Growth differentiation factor 15 | 163 | 107 | 3.95E-03 | -1.52 | 1.44 |
| 319162 | *Hist3h2a* | Histone H2A Type 3 (polyadenylated) | 55 | 40 | 3.42E-02 | -1.37 | -1.09 |
| 67311 | *Nanp* | N-acetylneuraminic acid phosphatase | 91 | 66 | 1.22E-02 | -1.38 | -1.63 |
| 18049 | *Ngf* | Nerve growth factor | 83 | 52 | 3.58E-02 | -1.6 | 1.27 |
| 21664 | *Phlda1* | Pleckstrin homology like domain | 52 | 32 | 1.84E-03 | -1.63 | 1.94 |
| 99890 | *Prmt6* | Protein arginine methyltransferase 6 | 125 | 98 | 4.02E-02 | -1.27 | 1.29 |
| 114872 | *Psg29* | Pregnancy-specific glycoprotein 29 | 536 | 263 | 1.52E-02 | -2.04 | 1.01 |
| 98710 | *Rabif* | RAB interacting factor | 84 | 74 | 3.16E-02 | -1.12 | -1.22 |
| 218215 | *Rnf144b* | Ring Finger Ubiquitin Ligase | 285 | 127 | 1.94E-02 | -2.25 | 2.07 |
| 20355 | *Sema4f* | Semaphorin 4F | 410 | 153 | 4.72E-02 | -2.68 | 3.03 |
| 75627 | *Snapc1* | Small nuclear RNA activating complex | 150 | 101 | 7.73E-03 | -1.49 | 1.01 |
| 21833 | *Thra* | Thyroid hormone receptor, alpha | 63 | 52 | 3.94E-02 | -1.22 | -1.35 |
| 380863 | *Tmem171* | Transmembrane protein | 132 | 115 | 2.42E-02 | -1.15 | 1.17 |
| 21929 | *Tnfaip3* | Zinc finger protein induced by TNF | 73 | 32 | 2.04E-02 | -2.29 | 1.91 |
| 319885 | *Zcchc7* | Zinc finger protein, role in RNA quality control | 110 | 77 | 2.75E-02 | -1.43 | 1.06 |

**Table S1: Twenty Four mRNAs are Destabilized in PARN Knockdown Cells**
