# Supplementary material for: The PARN Deadenylase Targets a Discrete Set of mRNAs for Decay and Regulates Cell Motility in Mouse Myoblasts
Source: PLoS Genet. 2012 Aug 30;8(8):e1002901. doi: 10.1371/journal.pgen.1002901 (PMC3431312; doi:10.1371/journal.pgen.1002901)
Supplement: Table S2 — Functional analysis of genes whose abundance was decreased >1.5-fold following PARN knockdown. (DOCX) [file pgen.1002901.s008.docx]

**Table S2: Functional Annotation Clustering of Gene Ontology terms Associated with Genes that are Down-Regulated in PARN Knockdown Cells.**

| **Cluster 1** | **Enrichment Score: 12.88** | | **Extracellular Matrix** | | | | |
| --- | --- | --- | --- | --- | --- | --- | --- |
| Category | Term | | Count | % | p-value | Fold Enrich | FDR |
| GO_CC_FAT | GO:0005576 | extracellular region | 114 | 15.53 | 1.05E-14 | 2.07 | 1.41E-11 |
| GO_CC_FAT | GO:0005578 | proteinaceous extracellular matrix | 42 | 5.72 | 2.68E-13 | 3.71 | 3.63E-10 |
| GO_CC_FAT | GO:0031012 | extracellular matrix | 43 | 5.86 | 2.76E-13 | 3.63 | 3.74E-10 |
| GO_CC_FAT | GO:0044421 | extracellular region part | 68 | 9.26 | 4.07E-13 | 2.59 | 5.52E-10 |
| **Cluster 2** | **Enrichment Score: 6.48** | | **Blood Vessel Development** | | | | |
| Category | Term | | Count | % | p-value | Fold Enrich | FDR |
| GO_BP_FAT | GO:0001568 | blood vessel development | 34 | 4.63 | 4.41E-09 | 3.20 | 7.71E-06 |
| GO_BP_FAT | GO:0001944 | vasculature development | 34 | 4.63 | 8.27E-09 | 3.13 | 1.45E-05 |
| GO_BP_FAT | GO:0048514 | blood vessel morphogenesis | 25 | 3.41 | 5.59E-06 | 2.86 | 9.77E-03 |
| GO_BP_FAT | GO:0001525 | angiogenesis | 18 | 2.45 | 5.73E-05 | 3.10 | 1.00E-01 |
| **Cluster 3** | **Enrichment Score: 4.03** | | **Neuron Development** | | | | |
| Category | Term | | Count | % | p-value | Fold Enrich | FDR |
| GO_BP_FAT | GO:0006928 | cell motion | 34 | 4.63 | 6.12E-06 | 2.36 | 1.07E-02 |
| GO_BP_FAT | GO:0007409 | axonogenesis | 20 | 2.72 | 1.66E-05 | 3.13 | 2.91E-02 |
| GO_BP_FAT | GO:0048667 | cell morphogenesis involved in neuron differentiation | 21 | 2.86 | 2.66E-05 | 2.93 | 4.65E-02 |
| GO_BP_FAT | GO:0000904 | cell morphogenesis involved in differentiation | 23 | 3.13 | 2.78E-05 | 2.75 | 4.85E-02 |
| GO_BP_FAT | GO:0007411 | axon guidance | 15 | 2.04 | 3.74E-05 | 3.72 | 6.54E-02 |
| GO_BP_FAT | GO:0032989 | cellular component morphogenesis | 32 | 4.36 | 4.35E-05 | 2.22 | 7.61E-02 |
| GO_BP_FAT | GO:0048812 | neuron projection morphogenesis | 20 | 2.72 | 5.15E-05 | 2.90 | 8.99E-02 |
| GO_BP_FAT | GO:0000902 | cell morphogenesis | 29 | 3.95 | 6.48E-05 | 2.28 | 1.13E-01 |
| GO_BP_FAT | GO:0031175 | neuron projection development | 22 | 3.00 | 1.31E-04 | 2.55 | 2.29E-01 |
| GO_BP_FAT | GO:0048858 | cell projection morphogenesis | 21 | 2.86 | 1.56E-04 | 2.59 | 2.73E-01 |
| GO_BP_FAT | GO:0032990 | cell part morphogenesis | 21 | 2.86 | 3.64E-04 | 2.43 | 6.35E-01 |
| GO_BP_FAT | GO:0030182 | neuron differentiation | 30 | 4.09 | 7.44E-04 | 1.94 | 1.29E+00 |
| GO_BP_FAT | GO:0048666 | neuron development | 24 | 3.27 | 1.13E-03 | 2.09 | 1.96E+00 |
| GO_BP_FAT | GO:0030030 | cell projection organization | 26 | 3.54 | 1.41E-03 | 1.98 | 2.43E+00 |
| **Cluster 4** | **Enrichment Score: 3.70** | | **Cell Movement** | | | | |
| Category | Term | | Count | % | p-value | Fold Enrich | FDR |
| GO_BP_FAT | GO:0006928 | cell motion | 34 | 4.63 | 6.12E-06 | 2.36 | 1.07E-02 |
| GO_BP_FAT | GO:0016477 | cell migration | 23 | 3.13 | 1.96E-04 | 2.42 | 3.41E-01 |
| GO_BP_FAT | GO:0051674 | localization of cell | 23 | 3.13 | 1.16E-03 | 2.12 | 2.01E+00 |
| GO_BP_FAT | GO:0048870 | cell motility | 23 | 3.13 | 1.16E-03 | 2.12 | 2.01E+00 |
| **Cluster 5** | **Enrichment Score: 3.55** | | **Lung Development** | | | | |
| Category | Term | | Count | % | p-value | Fold Enrich | FDR |
| GO_BP_FAT | GO:0035295 | tube development | 30 | 4.09 | 2.45E-06 | 2.66 | 4.29E-03 |
| GO_BP_FAT | GO:0060541 | respiratory system development | 16 | 2.18 | 1.39E-04 | 3.15 | 2.42E-01 |
| GO_BP_FAT | GO:0030324 | lung development | 14 | 1.91 | 6.01E-04 | 3.04 | 1.04E+00 |
| GO_BP_FAT | GO:0030323 | respiratory tube development | 14 | 1.91 | 6.71E-04 | 3.01 | 1.17E+00 |
| GO_BP_FAT | GO:0048286 | lung alveolus development | 5 | 0.68 | 1.25E-02 | 5.31 | 1.97E+01 |
| **Cluster 6** | **Enrichment Score: 3.50** | | **Tube Development** | | | | |
| Category | Term | | Count | % | p-value | Fold Enrich | FDR |
| GO_BP_FAT | GO:0035295 | tube development | 30 | 4.09 | 2.45E-06 | 2.66 | 4.29E-03 |
| GO_BP_FAT | GO:0002009 | morphogenesis of an epithelium | 21 | 2.86 | 2.97E-05 | 2.91 | 5.18E-02 |
| GO_BP_FAT | GO:0048729 | tissue morphogenesis | 25 | 3.41 | 5.00E-05 | 2.52 | 8.74E-02 |
| GO_BP_FAT | GO:0060429 | epithelium development | 26 | 3.54 | 9.37E-05 | 2.37 | 1.64E-01 |
| GO_BP_FAT | GO:0001763 | morphogenesis of a branching structure | 15 | 2.04 | 5.90E-04 | 2.90 | 1.03E+00 |
| GO_BP_FAT | GO:0048754 | branching morphogenesis of a tube | 13 | 1.77 | 6.66E-04 | 3.19 | 1.16E+00 |
| GO_BP_FAT | GO:0001656 | metanephros development | 9 | 1.23 | 2.35E-03 | 3.74 | 4.03E+00 |
| GO_BP_FAT | GO:0035239 | tube morphogenesis | 17 | 2.32 | 3.62E-03 | 2.24 | 6.15E+00 |
| GO_BP_FAT | GO:0060562 | epithelial tube morphogenesis | 11 | 1.50 | 2.77E-02 | 2.19 | 3.88E+01 |
| **Cluster 7** | **Enrichment Score: 3.10** | | **Receptor Protein Kinase Signaling** | | | | |
| Category | Term | | Count | % | p-value | Fold Enrich | FDR |
| GO_BP_FAT | GO:0007167 | enzyme linked receptor protein signaling pathway | 35 | 4.77 | 1.09E-08 | 3.03 | 1.91E-05 |
| GO_BP_FAT | GO:0007169 | transmembrane receptor protein tyrosine kinase signaling pathway | 26 | 3.54 | 3.52E-07 | 3.23 | 6.15E-04 |
| GO_MF_FAT | GO:0004714 | transmembrane receptor protein tyrosine kinase activity | 12 | 1.63 | 5.74E-06 | 5.55 | 8.67E-03 |
| GO_MF_FAT | GO:0004713 | protein tyrosine kinase activity | 16 | 2.18 | 2.12E-03 | 2.45 | 3.15E+00 |
| GO_BP_FAT | GO:0006468 | protein amino acid phosphorylation | 35 | 4.77 | 9.97E-02 | 1.29 | 8.41E+01 |
| GO_BP_FAT | GO:0016310 | phosphorylation | 38 | 5.18 | 1.38E-01 | 1.23 | 9.26E+01 |
| GO_MF_FAT | GO:0004672 | protein kinase activity | 29 | 3.95 | 3.00E-01 | 1.15 | 9.95E+01 |
| GO_MF_FAT | GO:0004674 | protein serine/threonine kinase activity | 15 | 2.04 | 8.95E-01 | 0.81 | 1.00E+02 |
| **Cluster 8** | **Enrichment Score: 3.05** | | **Bone Development** | | | | |
| Category | Term | | Count | % | p-value | Fold Enrich | FDR |
| GO_BP_FAT | GO:0001501 | skeletal system development | 28 | 3.81 | 6.82E-05 | 2.32 | 1.19E-01 |
| GO_BP_FAT | GO:0060348 | bone development | 14 | 1.91 | 1.02E-03 | 2.88 | 1.77E+00 |
| GO_BP_FAT | GO:0001503 | ossification | 11 | 1.50 | 1.00E-02 | 2.57 | 1.61E+01 |

| **Cluster 9** | **Enrichment Score: 2.95** | | | | **Carbohydrate Binding** | | | | | | | | |
| --- | --- | --- | --- | --- | --- | --- | --- | --- | --- | --- | --- | --- | --- |
| Category | Term | | | | Count | | | % | p-value | | Fold Enrich | | FDR |
| GO_MF_FAT | GO:0030246 | | carbohydrate binding | | 24 | | | 3.27 | 1.12E-04 | | 2.45 | | 1.69E-01 |
| GO_MF_FAT | GO:0001871 | | pattern binding | | 14 | | | 1.91 | 3.63E-04 | | 3.20 | | 5.47E-01 |
| GO_MF_FAT | GO:0030247 | | polysaccharide binding | | 14 | | | 1.91 | 3.63E-04 | | 3.20 | | 5.47E-01 |
| GO_MF_FAT | GO:0005539 | | glycosaminoglycan binding | | 12 | | | 1.63 | 1.58E-03 | | 3.08 | | 2.35E+00 |
| GO_MF_FAT | GO:0008201 | | heparin binding | | 7 | | | 0.95 | 7.27E-02 | | 2.37 | | 6.80E+01 |
| **Cluster 10** | **Enrichment Score: 2.41** | | | | **Regulation of Cell Adhesion** | | | | | | | | |
| Category | Term | | | | Count | | | % | p-value | | Fold Enrich | | FDR |
| GO_BP_FAT | GO:0030155 | | regulation of cell adhesion | | 13 | | | 1.77 | 3.57E-04 | | 3.41 | | 6.23E-01 |
| GO_BP_FAT | GO:0045785 | | positive regulation of cell adhesion | | 7 | | | 0.95 | 5.57E-03 | | 4.18 | | 9.30E+00 |
| GO_BP_FAT | GO:0010810 | | regulation of cell-substrate adhesion | | 7 | | | 0.95 | 6.52E-03 | | 4.06 | | 1.08E+01 |
| GO_BP_FAT | GO:0010811 | | positive regulation of cell-substrate adhesion | | 5 | | | 0.68 | 1.82E-02 | | 4.78 | | 2.75E+01 |
| **Cluster 11** | **Enrichment Score: 2.35** | | | | **Embryonic Development** | | | | | | | | |
| Category | Term | | | | Count | | | % | p-value | | Fold Enrich | | FDR |
| GO_BP_FAT | GO:0043009 | | chordate embryonic development | | 32 | | | 4.36 | 2.05E-03 | | 1.78 | | 3.53E+00 |
| GO_BP_FAT | GO:0048598 | | embryonic morphogenesis | | 28 | | | 3.81 | 2.36E-03 | | 1.85 | | 4.04E+00 |
| GO_BP_FAT | GO:0009792 | | embryonic development ending in birth or egg hatching | | 32 | | | 4.36 | 2.45E-03 | | 1.76 | | 4.20E+00 |
| GO_BP_FAT | GO:0001701 | | in utero embryonic development | | 21 | | | 2.86 | 9.52E-03 | | 1.85 | | 1.54E+01 |
| GO_BP_FAT | GO:0048568 | | embryonic organ development | | 18 | | | 2.45 | 1.50E-02 | | 1.88 | | 2.32E+01 |
| **Cluster 12** | **Enrichment Score: 2.35** | | | | **Kidney Development** | | | | | | | | |
| Category | Term | | | | Count | | | % | p-value | | Fold Enrich | | FDR |
| GO_BP_FAT | GO:0048754 | | branching morphogenesis of a tube | | 13 | | | 1.77 | 6.66E-04 | | 3.19 | | 1.16E+00 |
| GO_BP_FAT | GO:0001822 | | kidney development | | 13 | | | 1.77 | 1.30E-03 | | 2.96 | | 2.26E+00 |
| GO_BP_FAT | GO:0001657 | | ureteric bud development | | 8 | | | 1.09 | 1.59E-03 | | 4.50 | | 2.74E+00 |
| GO_BP_FAT | GO:0001655 | | urogenital system development | | 15 | | | 2.04 | 2.18E-03 | | 2.54 | | 3.75E+00 |
| GO_BP_FAT | GO:0001656 | | metanephros development | | 9 | | | 1.23 | 2.35E-03 | | 3.74 | | 4.03E+00 |
| GO_BP_FAT | GO:0060562 | | epithelial tube morphogenesis | | 11 | | | 1.50 | 2.77E-02 | | 2.19 | | 3.88E+01 |
| GO_BP_FAT | GO:0060675 | | ureteric bud morphogenesis | | 5 | | | 0.68 | 2.95E-02 | | 4.16 | | 4.07E+01 |
| GO_BP_FAT | GO:0001658 | | branching involved in ureteric bud morphogenesis | | 5 | | | 0.68 | 2.95E-02 | | 4.16 | | 4.07E+01 |
| **Cluster 13** | **Enrichment Score: 2.23** | | | | **Sterol Biosynthesis** | | | | | | | | |
| Category | Term | | | | Count | | | % | p-value | | Fold Enrich | | FDR |
| GO_BP_FAT | GO:0016126 | | sterol biosynthetic process | | 8 | | | 1.09 | 2.14E-04 | | 6.12 | | 3.73E-01 |
| GO_BP_FAT | GO:0006694 | | steroid biosynthetic process | | 10 | | | 1.36 | 5.22E-04 | | 4.16 | | 9.08E-01 |
| GO_BP_FAT | GO:0006695 | | cholesterol biosynthetic process | | 6 | | | 0.82 | 3.06E-03 | | 5.74 | | 5.22E+00 |
| GO_BP_FAT | GO:0008610 | | lipid biosynthetic process | | 22 | | | 3.00 | 4.38E-03 | | 1.95 | | 7.38E+00 |
| GO_BP_FAT | GO:0016125 | | sterol metabolic process | | 9 | | | 1.23 | 7.28E-03 | | 3.13 | | 1.20E+01 |
| GO_BP_FAT | GO:0008202 | | steroid metabolic process | | 12 | | | 1.63 | 1.71E-02 | | 2.25 | | 2.60E+01 |
| GO_BP_FAT | GO:0008203 | | cholesterol metabolic process | | 7 | | | 0.95 | 4.10E-02 | | 2.73 | | 5.19E+01 |
| GO_BP_FAT | GO:0008299 | | isoprenoid biosynthetic process | | 3 | | | 0.41 | 2.02E-01 | | 3.59 | | 9.81E+01 |
| **Cluster 14** | **Enrichment Score: 2.19** | | | | **Regulation of Cell Division and Growth** | | | | | | | | |
| Category | Term | | | | Count | | | % | p-value | | Fold Enrich | | FDR |
| GO_BP_FAT | GO:0051302 | | regulation of cell division | | 8 | | | 1.09 | 1.90E-03 | | 4.37 | | 3.27E+00 |
| GO_BP_FAT | GO:0051781 | | positive regulation of cell division | | 7 | | | 0.95 | 4.73E-03 | | 4.32 | | 7.96E+00 |
| GO_MF_FAT | GO:0008083 | | growth factor activity | | 12 | | | 1.63 | 1.01E-02 | | 2.42 | | 1.43E+01 |
| GO_BP_FAT | GO:0060688 | | regulation of morphogenesis of a branching structure | | 5 | | | 0.68 | 1.82E-02 | | 4.78 | | 2.75E+01 |
| **Cluster 15** | **Enrichment Score: 1.90** | | | | **Oxidoreductase Activity** | | | | | | | | |
| Category | Term | | | | Count | | | % | p-value | | Fold Enrich | | FDR |
| GO_MF_FAT | GO:0005507 | copper ion binding | | | 8 | | | 1.09 | 6.63E-03 | | 3.53 | | 9.57E+00 |
| GO_MF_FAT | GO:0016641 | oxidoreductase activity, acting on the CH-NH2 group of donors, oxygen as acceptor | | | 4 | | | 0.54 | 1.32E-02 | | 7.59 | | 1.82E+01 |
| GO_MF_FAT | GO:0016638 | oxidoreductase activity, acting on the CH-NH2 group of donors | | | 4 | | | 0.54 | 2.23E-02 | | 6.32 | | 2.89E+01 |
| **Cluster 16** | **Enrichment Score: 1.88** | | | | **Regulation of Cell Motion** | | | | | | | | |
| Category | Term | | | | Count | | % | | p-value | | Fold Enrich | | FDR |
| GO_BP_FAT | GO:0051270 | | | regulation of cell motion | 14 | 1.91 | | | 9.24E-04 | | 2.91 | | 1.60E+00 |
| GO_BP_FAT | GO:0030334 | | | regulation of cell migration | 12 | 1.63 | | | 2.05E-03 | | 2.98 | | 3.53E+00 |
| GO_BP_FAT | GO:0040012 | | | regulation of locomotion | 13 | 1.77 | | | 2.38E-03 | | 2.76 | | 4.08E+00 |
| GO_BP_FAT | GO:0030335 | | | positive regulation of cell migration | 5 | 0.68 | | | 8.32E-02 | | 2.99 | | 7.81E+01 |
| GO_BP_FAT | GO:0051272 | | | positive regulation of cell motion | 5 | 0.68 | | | 1.16E-01 | | 2.66 | | 8.85E+01 |
| GO_BP_FAT | GO:0040017 | | | positive regulation of locomotion | 5 | 0.68 | | | 1.16E-01 | | 2.66 | | 8.85E+01 |
| **Cluster 17** | **Enrichment Score: 1.75** | | | | **Germ Cell Development** | | | | | | | | |
| Category | Term | | | | Count | % | | | p-value | | Fold Enrich | | FDR |
| GO_BP_FAT | GO:0008354 | | | germ cell migration | 6 | 0.82 | | | 7.69E-05 | | 11.47 | | 1.34E-01 |
| GO_BP_FAT | GO:0048610 | | | reproductive cellular process | 10 | 1.36 | | | 2.05E-01 | | 1.53 | | 9.82E+01 |
| GO_BP_FAT | GO:0007281 | | | germ cell development | 6 | 0.82 | | | 3.54E-01 | | 1.53 | | 1.00E+02 |
| **Cluster 18** | **Enrichment Score: 1.70** | | | | **Face and Head Development** | | | | | | | | |
| Category | Term | | | | Count | % | | | p-value | | Fold Enrich | | FDR |
| GO_BP_FAT | GO:0060324 | | | face development | 5 | 0.68 | | | 4.83E-03 | | 6.83 | | 8.11E+00 |
| GO_BP_FAT | GO:0060322 | | | head development | 5 | 0.68 | | | 1.25E-02 | | 5.31 | | 1.97E+01 |
| GO_BP_FAT | GO:0060325 | | | face morphogenesis | 4 | | | 0.54 | | 2.18E-02 | 6.37 | | 3.20E+01 |
| GO_BP_FAT | GO:0060323 | | | head morphogenesis | 4 | | | 0.54 | | 3.35E-02 | | 5.46 | 4.48E+01 |
| GO_BP_FAT | GO:0010171 | | | body morphogenesis | 4 | | | 0.54 | | 7.36E-02 | | 4.03 | 7.37E+01 |

| **Cluster 19** | **Enrichment Score: 1.67** | | **Response to Stimulus** | | | | |
| --- | --- | --- | --- | --- | --- | --- | --- |
| Category | Term | | Count | % | p-value | Fold Enrich | FDR |
| GO_BP_FAT | GO:0009725 | response to hormone stimulus | 14 | 1.91 | 6.81E-03 | 2.33 | 1.13E+01 |
| GO_BP_FAT | GO:0009719 | response to endogenous stimulus | 15 | 2.04 | 8.30E-03 | 2.19 | 1.36E+01 |
| GO_BP_FAT | GO:0010033 | response to organic substance | 22 | 3.00 | 1.72E-01 | 1.31 | 9.63E+01 |
| **Cluster 20** | **Enrichment Score: 1.66** | | **Regulation of Development** | | | | |
| Category | Term | | Count | % | p-value | Fold Enrich | FDR |
| GO_BP_FAT | GO:0060284 | regulation of cell development | 19 | 2.59 | 1.10E-04 | 2.84 | 1.92E-01 |
| GO_BP_FAT | GO:0045596 | negative regulation of cell differentiation | 19 | 2.59 | 1.17E-03 | 2.34 | 2.03E+00 |
| GO_BP_FAT | GO:0010769 | regulation of cell morphogenesis involved in differentiation | 9 | 1.23 | 1.28E-03 | 4.10 | 2.21E+00 |
| GO_BP_FAT | GO:0010721 | negative regulation of cell development | 8 | 1.09 | 1.90E-03 | 4.37 | 3.27E+00 |
| GO_BP_FAT | GO:0031344 | regulation of cell projection organization | 9 | 1.23 | 2.70E-03 | 3.66 | 4.62E+00 |
| GO_BP_FAT | GO:0051129 | negative regulation of cellular component organization | 12 | 1.63 | 3.74E-03 | 2.76 | 6.34E+00 |
| GO_BP_FAT | GO:0050768 | negative regulation of neurogenesis | 7 | 0.95 | 6.52E-03 | 4.06 | 1.08E+01 |
| GO_BP_FAT | GO:0050770 | regulation of axonogenesis | 7 | 0.95 | 7.57E-03 | 3.94 | 1.24E+01 |
| GO_BP_FAT | GO:0050771 | negative regulation of axonogenesis | 5 | 0.68 | 8.08E-03 | 5.98 | 1.32E+01 |
| GO_BP_FAT | GO:0051960 | regulation of nervous system development | 14 | 1.91 | 9.01E-03 | 2.25 | 1.46E+01 |
| GO_BP_FAT | GO:0050767 | regulation of neurogenesis | 13 | 1.77 | 9.03E-03 | 2.35 | 1.47E+01 |
| GO_BP_FAT | GO:0045664 | regulation of neuron differentiation | 11 | 1.50 | 9.19E-03 | 2.60 | 1.49E+01 |
| GO_BP_FAT | GO:0048640 | negative regulation of developmental growth | 4 | 0.54 | 1.29E-02 | 7.65 | 2.03E+01 |
| GO_BP_FAT | GO:0010975 | regulation of neuron projection development | 7 | 0.95 | 1.30E-02 | 3.52 | 2.05E+01 |
| GO_BP_FAT | GO:0048638 | regulation of developmental growth | 6 | 0.82 | 1.59E-02 | 3.96 | 2.45E+01 |
| GO_BP_FAT | GO:0031345 | negative regulation of cell projection organization | 5 | 0.68 | 1.82E-02 | 4.78 | 2.75E+01 |
| GO_BP_FAT | GO:0022604 | regulation of cell morphogenesis | 10 | 1.36 | 3.05E-02 | 2.28 | 4.18E+01 |
| GO_BP_FAT | GO:0032535 | regulation of cellular component size | 13 | 1.77 | 5.92E-02 | 1.79 | 6.56E+01 |
| GO_BP_FAT | GO:0030517 | negative regulation of axon extension | 3 | 0.41 | 6.18E-02 | 7.17 | 6.72E+01 |
| GO_BP_FAT | GO:0031346 | positive regulation of cell projection organization | 4 | 0.54 | 9.36E-02 | 3.64 | 8.21E+01 |
| GO_BP_FAT | GO:0008361 | regulation of cell size | 9 | 1.23 | 1.12E-01 | 1.85 | 8.74E+01 |
| GO_BP_FAT | GO:0010720 | positive regulation of cell development | 5 | 0.68 | 1.54E-01 | 2.39 | 9.46E+01 |
| GO_BP_FAT | GO:0030516 | regulation of axon extension | 3 | 0.41 | 1.64E-01 | 4.10 | 9.56E+01 |
| GO_BP_FAT | GO:0030308 | negative regulation of cell growth | 5 | 0.68 | 1.64E-01 | 2.33 | 9.56E+01 |
| GO_BP_FAT | GO:0045597 | positive regulation of cell differentiation | 11 | 1.50 | 1.78E-01 | 1.54 | 9.68E+01 |
| GO_BP_FAT | GO:0045926 | negative regulation of growth | 6 | 0.82 | 1.93E-01 | 1.94 | 9.77E+01 |
| GO_BP_FAT | GO:0001558 | regulation of cell growth | 7 | 0.95 | 2.05E-01 | 1.76 | 9.82E+01 |
| GO_BP_FAT | GO:0045792 | negative regulation of cell size | 5 | 0.68 | 2.18E-01 | 2.08 | 9.86E+01 |
| GO_BP_FAT | GO:0040008 | regulation of growth | 14 | 1.91 | 2.51E-01 | 1.33 | 9.94E+01 |
| GO_BP_FAT | GO:0050769 | positive regulation of neurogenesis | 4 | 0.54 | 2.61E-01 | 2.25 | 9.95E+01 |
| **Cluster 21** | **Enrichment Score: 1.62** | | **Collagen** | | | | |
| Category | Term | | Count | % | p-value | Fold Enrich | FDR |
| GO_MF_FAT | GO:0048407 | platelet-derived growth factor binding | 5 | 0.68 | 7.72E-04 | 10.54 | 1.16E+00 |
| GO_CC_FAT | GO:0005583 | fibrillar collagen | 3 | 0.41 | 4.11E-02 | 8.82 | 4.34E+01 |
| GO_CC_FAT | GO:0005581 | collagen | 4 | 0.54 | 6.77E-02 | 4.15 | 6.13E+01 |
| GO_MF_FAT | GO:0005201 | extracellular matrix structural constituent | 4 | 0.54 | 1.54E-01 | 2.92 | 9.21E+01 |
| **Cluster 22** | **Enrichment Score: 1.60** | | **Mesenchymal Development** | | | | |
| Category | Term | | Count | % | p-value | Fold Enrich | FDR |
| GO_BP_FAT | GO:0001667 | ameboidal cell migration | 7 | 0.95 | 1.00E-02 | 3.72 | 1.62E+01 |
| GO_BP_FAT | GO:0014031 | mesenchymal cell development | 7 | 0.95 | 1.15E-02 | 3.62 | 1.83E+01 |
| GO_BP_FAT | GO:0048762 | mesenchymal cell differentiation | 7 | 0.95 | 1.30E-02 | 3.52 | 2.05E+01 |
| GO_BP_FAT | GO:0060485 | mesenchyme development | 7 | 0.95 | 1.48E-02 | 3.43 | 2.29E+01 |
| GO_BP_FAT | GO:0014032 | neural crest cell development | 5 | 0.68 | 3.39E-02 | 3.98 | 4.53E+01 |
| GO_BP_FAT | GO:0014033 | neural crest cell differentiation | 5 | 0.68 | 3.39E-02 | 3.98 | 4.53E+01 |
| GO_BP_FAT | GO:0001755 | neural crest cell migration | 3 | 0.41 | 2.41E-01 | 3.19 | 9.92E+01 |
| **Cluster 23** | **Enrichment Score: 1.36** | | **Development** | | | | |
| Category | Term | | Count | % | p-value | Fold Enrich | FDR |
| GO_BP_FAT | GO:0060021 | palate development | 9 | 1.23 | 1.80E-04 | 5.38 | 3.14E-01 |
| GO_BP_FAT | GO:0048705 | skeletal system morphogenesis | 16 | 2.18 | 6.84E-04 | 2.73 | 1.19E+00 |
| GO_BP_FAT | GO:0048701 | embryonic cranial skeleton morphogenesis | 6 | 0.82 | 4.77E-03 | 5.21 | 8.02E+00 |
| GO_BP_FAT | GO:0048568 | embryonic organ development | 18 | 2.45 | 1.50E-02 | 1.88 | 2.32E+01 |
| GO_BP_FAT | GO:0048562 | embryonic organ morphogenesis | 12 | 1.63 | 5.36E-02 | 1.88 | 6.18E+01 |
| GO_BP_FAT | GO:0048704 | embryonic skeletal system morphogenesis | 7 | 0.95 | 7.04E-02 | 2.39 | 7.21E+01 |
| GO_BP_FAT | GO:0042474 | middle ear morphogenesis | 3 | 0.41 | 1.45E-01 | 4.41 | 9.36E+01 |
| GO_BP_FAT | GO:0001654 | eye development | 9 | 1.23 | 1.63E-01 | 1.69 | 9.55E+01 |
| GO_BP_FAT | GO:0048706 | embryonic skeletal system development | 7 | 0.95 | 1.65E-01 | 1.89 | 9.57E+01 |
| GO_BP_FAT | GO:0043010 | camera-type eye development | 8 | 1.09 | 1.68E-01 | 1.76 | 9.60E+01 |
| GO_BP_FAT | GO:0007423 | sensory organ development | 12 | 1.63 | 2.81E-01 | 1.34 | 9.97E+01 |
| GO_BP_FAT | GO:0043583 | ear development | 5 | 0.68 | 5.12E-01 | 1.35 | 1.00E+02 |
| GO_BP_FAT | GO:0042471 | ear morphogenesis | 3 | 0.41 | 7.63E-01 | 1.10 | 1.00E+02 |
| **Cluster 24** | **Enrichment Score: 1.34** | | **Limb Development** | | | | |
| Category | Term | | Count | % | p-value | Fold Enrich | FDR |
| GO_BP_FAT | GO:0048598 | embryonic morphogenesis | 28 | 3.81 | 2.36E-03 | 1.85 | 4.04E+00 |
| GO_BP_FAT | GO:0030326 | embryonic limb morphogenesis | 10 | 1.36 | 4.20E-02 | 2.15 | 5.28E+01 |
| GO_BP_FAT | GO:0035113 | embryonic appendage morphogenesis | 10 | 1.36 | 4.20E-02 | 2.15 | 5.28E+01 |
| GO_BP_FAT | GO:0035108 | limb morphogenesis | 10 | 1.36 | 6.95E-02 | 1.95 | 7.16E+01 |
| GO_BP_FAT | GO:0035107 | appendage morphogenesis | 10 | 1.36 | 6.95E-02 | 1.95 | 7.16E+01 |
| GO_BP_FAT | GO:0060173 | limb development | 10 | 1.36 | 7.31E-02 | 1.93 | 7.35E+01 |
| GO_BP_FAT | GO:0048736 | appendage development | 10 | 1.36 | 7.31E-02 | 1.93 | 7.35E+01 |
| **Cluster 25** | **Enrichment Score: 1.32** | | **Fatty Acid Biosynthesis** | | | | |
| Category | Term | | Count | % | p-value | Fold Enrich | FDR |
| GO_BP_FAT | GO:0006631 | fatty acid metabolic process | 16 | 2.18 | 3.88E-03 | 2.30 | 6.57E+00 |
| GO_BP_FAT | GO:0046394 | carboxylic acid biosynthetic process | 11 | 1.50 | 4.96E-02 | 1.98 | 5.89E+01 |
| GO_BP_FAT | GO:0016053 | organic acid biosynthetic process | 11 | 1.50 | 4.96E-02 | 1.98 | 5.89E+01 |
| GO_BP_FAT | GO:0006633 | fatty acid biosynthetic process | 7 | 0.95 | 8.60E-02 | 2.27 | 7.92E+01 |
| GO_BP_FAT | GO:0005506 | iron ion binding | 15 | 2.04 | 2.97E-01 | 1.26 | 9.95E+01 |

Gene IDs of those transcripts that were down-regulated 1.5 fold or more in PARN KD cells were uploaded to DAVID along with all genes in the dataset as a background list. Functional annotation clustering of the enriched GO-FAT terms for biological process (GO_BP_FAT), cellular component (GO_CC_FAT) and molecular function (GO_MF_FAT) was performed on 734 Gene IDs using Medium Classification. The twenty-five clusters with Enrichment Scores >.1.3 are shown and were annotated manually. “Count” refers to the number of genes in the dataset associated with each GO term; “%” refers to the percent of genes in the up-regulated dataset associated with each GO term. The p-value is the EASE Score/ modified Fisher’s exact P-value (p<0.05 is considered significant). FDR is the false discovery rate (FDR<5.0 is considered significant).
