# Supplementary material for: The PARN Deadenylase Targets a Discrete Set of mRNAs for Decay and Regulates Cell Motility in Mouse Myoblasts
Source: PLoS Genet. 2012 Aug 30;8(8):e1002901. doi: 10.1371/journal.pgen.1002901 (PMC3431312; doi:10.1371/journal.pgen.1002901)
Supplement: Table S3 — Functional analysis of genes whose abundance was increased >1.5-fold following PARN knockdown. (DOCX) [file pgen.1002901.s009.docx]

**Table S3: Functional Annotation Clustering of Gene Ontology terms Associated with Genes that are Up-Regulated in PARN Knockdown Cells.**

| **Cluster 1** | **Enrichment Score: 10.98** | | **Ribosome/Translation** | | | | |
| --- | --- | --- | --- | --- | --- | --- | --- |
| Category | Term |  | Count | % | p-value | Fold Enrich | FDR |
| GO_CC_FAT | GO:0030529 | ribonucleoprotein complex | 41 | 12.20 | 1.16E-12 | 3.63 | 1.51E-09 |
| GO_MF_FAT | GO:0003735 | structural constituent of ribosome | 23 | 6.85 | 1.43E-12 | 6.88 | 1.95E-09 |
| GO_MF_FAT | GO:0005198 | structural molecule activity | 34 | 10.12 | 3.18E-12 | 4.21 | 4.31E-09 |
| GO_CC_FAT | GO:0005840 | ribosome | 25 | 7.44 | 3.32E-11 | 5.32 | 4.29E-08 |
| GO_BP_FAT | GO:0006412 | translation | 27 | 8.04 | 7.12E-10 | 4.26 | 1.14E-06 |
| **Cluster 2** | **Enrichment Score: 7.92** | | **Non-Membrane-Bounded Organelle** | | | | |
| Category | Term |  | Count | % | p-value | Fold Enrich | FDR |
| GO_MF_FAT | GO:0005198 | structural molecule activity | 34 | 10.12 | 3.18E-12 | 4.21 | 4.31E-09 |
| GO_CC_FAT | GO:0043232 | intracellular non-membrane-bounded organelle | 74 | 22.02 | 7.37E-07 | 1.73 | 9.54E-04 |
| GO_CC_FAT | GO:0043228 | non-membrane-bounded organelle | 74 | 22.02 | 7.37E-07 | 1.73 | 9.54E-04 |
| **Cluster 3** | **Enrichment Score: 1.95** | | **Muscle Process** | | | | |
| Category | Term |  | Count | % | p-value | Fold Enrich | FDR |
| GO_CC_FAT | GO:0005865 | striated muscle thin filament | 4 | 1.19 | 5.44E-04 | 22.13 | 7.01E-01 |
| GO_CC_FAT | GO:0030017 | sarcomere | 7 | 2.08 | 7.30E-03 | 4.05 | 9.05E+00 |
| GO_CC_FAT | GO:0044449 | contractile fiber part | 7 | 2.08 | 1.03E-02 | 3.76 | 1.25E+01 |
| GO_CC_FAT | GO:0030016 | myofibril | 7 | 2.08 | 1.32E-02 | 3.57 | 1.58E+01 |
| GO_CC_FAT | GO:0043292 | contractile fiber | 7 | 2.08 | 1.58E-02 | 3.43 | 1.86E+01 |
| GO_BP_FAT | GO:0006936 | muscle contraction | 5 | 1.49 | 1.68E-02 | 5.02 | 2.38E+01 |
| GO_BP_FAT | GO:0003012 | muscle system process | 5 | 1.49 | 2.40E-02 | 4.51 | 3.24E+01 |
| GO_CC_FAT | GO:0015629 | actin cytoskeleton | 9 | 2.68 | 6.98E-02 | 2.06 | 6.08E+01 |
| **Cluster 4** | **Enrichment Score: 1.92** | | **Nucleolus** | | | | |
| Category | Term |  | Count | % | p-value | Fold Enrich | FDR |
| GO_CC_FAT | GO:0005730 | nucleolus | 20 | 5.95 | 2.35E-04 | 2.60 | 3.04E-01 |
| GO_CC_FAT | GO:0031974 | membrane-enclosed lumen | 41 | 12.20 | 8.67E-03 | 1.49 | 1.07E+01 |
| GO_CC_FAT | GO:0043233 | organelle lumen | 37 | 11.01 | 3.58E-02 | 1.38 | 3.76E+01 |
| GO_CC_FAT | GO:0070013 | intracellular organelle lumen | 36 | 10.71 | 5.27E-02 | 1.35 | 5.04E+01 |
| GO_CC_FAT | GO:0031981 | nuclear lumen | 29 | 8.63 | 6.19E-02 | 1.39 | 5.63E+01 |
| **Cluster 5** | **Enrichment Score: 1.71** | | **Mitochondrion** | | | | |
| Category | Term |  | Count | % | p-value | Fold Enrich | FDR |
| GO_CC_FAT | GO:0044429 | mitochondrial part | 25 | 7.44 | 8.21E-04 | 2.09 | 1.06E+00 |
| GO_CC_FAT | GO:0005740 | mitochondrial envelope | 18 | 5.36 | 6.79E-03 | 2.04 | 8.44E+00 |
| GO_CC_FAT | GO:0031966 | mitochondrial membrane | 17 | 5.06 | 9.34E-03 | 2.03 | 1.14E+01 |
| GO_CC_FAT | GO:0005739 | mitochondrion | 42 | 12.50 | 1.76E-02 | 1.41 | 2.05E+01 |
| GO_CC_FAT | GO:0031967 | organelle envelope | 21 | 6.25 | 2.01E-02 | 1.71 | 2.31E+01 |
| GO_CC_FAT | GO:0031975 | envelope | 21 | 6.25 | 2.10E-02 | 1.70 | 2.40E+01 |
| GO_CC_FAT | GO:0005743 | mitochondrial inner membrane | 13 | 3.87 | 3.97E-02 | 1.91 | 4.08E+01 |
| GO_CC_FAT | GO:0019866 | organelle inner membrane | 13 | 3.87 | 5.72E-02 | 1.80 | 5.34E+01 |
| GO_CC_FAT | GO:0031090 | organelle membrane | 20 | 5.95 | 4.55E-01 | 1.10 | 1.00E+02 |
| **Cluster 6** | **Enrichment Score: 1.69** | | **RNA Processing** | | | | |
| Category | Term |  | Count | % | p-value | Fold Enrich | FDR |
| GO_CC_FAT | GO:0005681 | spliceosome | 10 | 2.98 | 4.12E-03 | 3.17 | 5.20E+00 |
| GO_BP_FAT | GO:0008380 | RNA splicing | 11 | 3.27 | 1.31E-02 | 2.48 | 1.91E+01 |
| GO_BP_FAT | GO:0006397 | mRNA processing | 12 | 3.57 | 2.45E-02 | 2.14 | 3.29E+01 |
| GO_BP_FAT | GO:0006396 | RNA processing | 16 | 4.76 | 4.63E-02 | 1.71 | 5.33E+01 |
| GO_BP_FAT | GO:0016071 | mRNA metabolic process | 12 | 3.57 | 5.81E-02 | 1.85 | 6.18E+01 |
| **Cluster 7** | **Enrichment Score: 1.40** | | **Muscle Development** | | | | |
| Category | Term |  | Count | % | p-value | Fold Enrich | FDR |
| GO_BP_FAT | GO:0007519 | skeletal muscle tissue development | 7 | 2.08 | 2.03E-03 | 5.24 | 3.21E+00 |
| GO_BP_FAT | GO:0060538 | skeletal muscle organ development | 7 | 2.08 | 2.21E-03 | 5.16 | 3.49E+00 |
| GO_BP_FAT | GO:0014706 | striated muscle tissue development | 8 | 2.38 | 9.89E-03 | 3.34 | 1.48E+01 |
| GO_MF_FAT | GO:0008307 | structural constituent of muscle | 3 | 0.89 | 1.37E-02 | 16.05 | 1.71E+01 |
| GO_BP_FAT | GO:0060537 | muscle tissue development | 8 | 2.38 | 1.50E-02 | 3.08 | 2.16E+01 |
| GO_BP_FAT | GO:0007517 | muscle organ development | 9 | 2.68 | 1.86E-02 | 2.69 | 2.60E+01 |
| GO_BP_FAT | GO:0055002 | striated muscle cell development | 4 | 1.19 | 6.81E-02 | 4.21 | 6.78E+01 |
| GO_BP_FAT | GO:0055001 | muscle cell development | 4 | 1.19 | 9.32E-02 | 3.68 | 7.92E+01 |
| GO_BP_FAT | GO:0048741 | skeletal muscle fiber development | 3 | 0.89 | 1.08E-01 | 5.30 | 8.41E+01 |
| GO_BP_FAT | GO:0048747 | muscle fiber development | 3 | 0.89 | 1.46E-01 | 4.42 | 9.20E+01 |
| GO_BP_FAT | GO:0051146 | striated muscle cell differentiation | 4 | 1.19 | 2.21E-01 | 2.46 | 9.82E+01 |
| GO_BP_FAT | GO:0042692 | muscle cell differentiation | 4 | 1.19 | 3.74E-01 | 1.82 | 9.99E+01 |
| GO_BP_FAT | GO:0043062 | extracellular structure organization | 4 | 1.19 | 4.87E-01 | 1.52 | 1.00E+02 |

Gene IDs of those transcripts that were up-regulated 1.5 fold or more in PARN KD cells were uploaded to DAVID along with all genes in the dataset as a background list. Functional annotation clustering of the enriched GO-FAT terms for biological process (GO_BP_FAT), cellular component (GO_CC_FAT) and molecular function (GO_MF_FAT) was performed on 336 Gene IDs using Medium Classification. The seven clusters with Enrichment Scores >1.3 are shown. “Count” refers to the number of genes in the dataset associated with each GO term; “%” refers to the percent of genes in the up-regulated dataset associated with each GO term. The p-value is the EASE Score/ modified Fisher’s exact P-value (p<0.05 is considered significant). FDR is the false discovery rate (FDR<5.0 is considered significant).
