# Supplementary material for: The PARN Deadenylase Targets a Discrete Set of mRNAs for Decay and Regulates Cell Motility in Mouse Myoblasts
Source: PLoS Genet. 2012 Aug 30;8(8):e1002901. doi: 10.1371/journal.pgen.1002901 (PMC3431312; doi:10.1371/journal.pgen.1002901)
Supplement: Table S4 — Gene Sequence Information. (DOCX) [file pgen.1002901.s010.docx]

**Table S4: Gene Sequence Information**

| **A: Sequences of Oligonucleotides Used for qRT-PCR** | | | | |
| --- | --- | --- | --- | --- |
| Gene ID | Gene Name | Forward Primer | | Reverse Primer |
| 103948 | *7SL* *RNA** | GGAGTTCTGGGCTGTAGTGC | | ATCAGCACGGGAGTTTTGAC |
| 109711 | *Actb* | AGAGGGAAATCGTGCGTGAC | | CAATAGTGATGACCTGGCCGT |
| 11541 | *Adora2b* | TGCTCACACAGAGCTCCATC | | TGTCCCAGTGACCAAACCTT |
| 11541 | *Adora2b* (pre-mRNA) | AAACCTCCAAGGCTAAAGGC | | TGTCCCAGTGACCAAACCTT |
| 11689 | *Alox5* | GTAAAGAACTGGAGGCACGG | | ATATCTCGGGGCAGATCCTT |
| 223690 | *Ankrd54* | CAATGCCAACGACGTAGAAA | | ATTGCAGGAAGCAAAATGGA |
| 223690 | *Ankrd54* (pre-mRNA) | CAGCATAGCTAACACGCCTG | | ATTGCAGGAAGCAAAATGGA |
| 12111 | *Bgn* | ATTGCCCTACCCAGAACTTGAC | | GCAGAGTATGAACCCTTTCCTG |
| 12319 | *Car8* | ATGGCTGACCTGAGCTTCATT | | ACCTTCCTCGTAACCCCACT |
| 12490 | *Cd34* | AAGGCTGGGTGAAGACCCTTA | | TGAATGGCCGTTTCTGGAAGT |
| 12763 | *Cmah* | CTGTCCGTTTGCTGGGTACT | | TGTTGAGCTGATTTGGGTCA |
| 12842 | *Col1a1* | CCAAGAAGACATCCCTGAAGTCA | | TGCACGTCATCGCACACA |
| 12483 | *Col1a2* | AGCTTTGTGGATACGCGGACT | | TCGTACTGCTCCCGATTCCA |
| 12833 | *Col6a1* | TACTTCGGGAAAGGCACCTA | | TCGGTCACCACGATCAAGTA |
| 66362 | *Exosc3* | GCATTTGAAGGGGCAACTAA | | TCAATGCAGACCATCTCTGG |
| 14178 | *Fgf7* | CTCTACAGGTCATGCTTCCACC | | ACAGAACAGTCTTCTCACCCT |
| 14433 | *Gapdh* | TCACCACCATGGAGAAGGC | | GCTAAGCAGTTGGTGGTGCA |
| 67839 | *Gpsm1* | CTGACTAGCCCAGCAGCAG | | TCTCGGCACTCAGCCTCT |
| 67839 | *Gpsm1* (pre-mRNA) | CTGCTGAGTCCTTGGTCTCC | | TCTCGGCACTCAGCCTCT |
| 16773 | *Lama2* | GAAGGCTCCCAGACTCAGC | | CCCACATGTAGCATTGGTTG |
| 17260 | *Mef2c* | ACGAGGATAATGGATGAGCGT | | ATCAGTGCAATCTCACAGTCG |
| 18039 | *Nefl* | TGCAGGCTCGCTATGAGGA | | CAGGAAAGCTATCTCGTCCATC |
| 74108 | *Parn* | CTCAGCCAGCCAGAACAAGT | | TGCTTGCCTTTCTGCTTCTT |
| 12193 | *Zfp36l2* | CCTCCTTTGTGGTGGTTGTT | | ACACTACGTGGTGGCAATGA |
| 12193 | *Zfp36l2* (pre-mRNA) | CACCAGAAACCCCAACAAGT | | TGTCCAGCATGTTGTTCAGA |
| * We used primers described previously (Misra *et al* (2005) *J Biol Chem* 280: 29364–29373) | | | | |
| **B: Accession Numbers of Genes Mentioned in the Text** | | | |  |
| Gene ID | Gene Name | Gene ID | Gene Name |  |
| 68737 | *Angel1* | 14281 | *Fos* |  |
| 52477 | *Angel2* | 11991 | *Hnrnpd/Auf1* |  |
| 12457 | *Ccrn4l* | 140486 | *Igf2bp1/Imp1/Zbp1* |  |
| 13046 | *Celf1/Cugbp1* | 319765 | *Igf2bp2* |  |
| 104625 | *Cnot6* | 17869 | *Myc* |  |
| 231464 | *Cnot6l* | 103135 | *Pan2* |  |
| 18983 | *Cnot7* | 80193 | *Pum2* |  |
| 69125 | *Cnot8* | 233833 | *Tnrc6a/GW182* |  |
| 12877 | *Cpeb1* | 24127 | *Xrn1* |  |
| 15568 | *Elavl1/HuR* | 22695 | *Zfp36/Ttp/Tis11* |  |
